# Safety and Efficacy of Imatinib for Hospitalized Adults with COVID-19: A structured summary of a study protocol for a randomised controlled trial

**DOI:** 10.1186/s13063-020-04819-9

**Published:** 2020-10-28

**Authors:** Ashkan Emadi, Joel V. Chua, Rohit Talwani, Søren M. Bentzen, John Baddley

**Affiliations:** 1 University of Maryland Marlene and Stewart Greenebaum Comprehensive Cancer Center, 22 South Greene Street, Room N9E06, Baltimore, Maryland 21201 USA; 2 University of Maryland School of Medicine Department of Medicine, Baltimore, MD USA; 3 University of Maryland School of Medicine Department of Pharmacology, Baltimore, MD USA; 4 University of Maryland School of Medicine, Institute of Human Virology, Baltimore, USA; 5 University of Maryland School of Medicine Department of Epidemiology and Public Health, Baltimore, MD USA

**Keywords:** COVID-19, Randomised controlled trial, protocol, SARS-CoV, imatinib, tyrosine kinase inhibitor, TKI, lysosomal sequestration, viral ABL-1 signalling, TKI-induced attenuation of vascular leak, TKI-induced prevention and treatment lung inflammation

## Abstract

**Objectives:**

Primary Objective: To evaluate the efficacy and safety of oral administration of imatinib combined with the Best Conventional Care (BCC) versus placebo plus BCC in hospitalized patients with COVID-19.

Hypothesis: Addition of imatinib to the BCC will provide a superior clinical outcome for patients with COVID-19 compared with BCC plus placebo.

This hypothesis is on the basis of 1) intralysosomal entrapment of imatinib will increase endosomal pH and effectively decrease SARS-CoV-2/cell fusion, 2) kinase inhibitory activity of imatinib will interfere with budding/release or replication of SARS-CoV-2, and 3) because of the critical role of mechanical ventilation in the care of patients with ARDS, imatinib will have a significant clinical impact for patients with critical COVID-19 infection in Intensive Care Unit (ICU).

**Trial design:**

This is an individual patient-level randomized, double-blind, placebo-controlled, two-parallel arm phase 3 study to evaluate the safety and efficacy of imatinib for the treatment of hospitalized adults with COVID-19. Participants will be followed for up to 60 days from the start of study drug administration. This trial will be conducted in accordance with the principles of the Declaration of Helsinki and the Good Clinical Practice guidelines of the International Conference on Harmonization.

**Participants:**

*Inclusion Criteria*: Patients may be included in the study only if they meet all of the following criteria: 1) Ability to understand and willingness to sign a written informed consent document. Informed consent must be obtained prior to participation in the study. For patients who are too unwell to provide consent such as patients on invasive ventilator or extracorporeal membrane oxygenation (ECMO), their Legally Authorized Representative (LAR) can sign the informed consent, 2) Hospitalized patients ≥18 years of age, 3) Positive reverse transcriptase-polymerase chain reaction (RT-PCR) assay for SARS-CoV-2 in the respiratory tract sample (oropharyngeal, nasopharyngeal or bronchoalveolar lavage (BAL)) by Center for Disease Control or local laboratory within 7 days of randomization, 4) Women of childbearing potential must agree to use at least one primary form of contraception for the duration of the study.

*Exclusion Criteria*: Patients meeting any of the following criteria are not eligible for the study: 1) Patients receiving any other investigational agents in a clinical trial. Off-label use of agents such as hydroxychloroquine is not an exclusion criterion, 2) Pregnant or breastfeeding women, 3) Patients with significant liver or renal dysfunction at the time of screening as defined as: 3.1) Direct bilirubin >2.5 mg/dL, 3.2) AST, ALT, or alkaline phosphatase >5x upper limit of normal, 3.3) eGFR ≤30 mL/min or requiring renal replacement therapy, 4) Patients with significant hematologic disorder at screen as defined as: 4.1) Absolute neutrophil count (ANC) <500/μL, 4.2) Platelet <20,000/μL, 4.3) Hemoglobin <7 g/dL, 5) Uncontrolled underlying illness including, but not limited to, symptomatic congestive heart failure, unstable angina pectoris, uncontrolled active seizure disorder, or psychiatric illness/social situations that per site Principal Investigator’s judgment would limit compliance with study requirements, 6) Known allergy to imatinib or its component products, 7) Any other clinical conditions that in the opinion of the investigator would make the subject unsuitable for the study.

Both men and women of all races and ethnic groups are eligible for this trial.

University of Maryland Medical Center, Baltimore, MD is the initiating site. The study may be opened in other centers on the basis of the accrual rate or the magnitude of the COVID-19 pandemic.

**Intervention and comparator:**

*Imatinib*: All doses of imatinib should be administered with a meal and a large glass of water. Imatinib can be dissolved in water or apple juice for patients having difficulty swallowing. In this study, patients with confirmed positive COVID-19 tests receive imatinib for a total of 14 days; 400 mg orally daily Days 1-14. Imatinib 400 mg tablets will be encapsulated using size 000 capsules and cellulose microcrystalline filler. For patients on ventilator or ECMO, imatinib will be given as oral suspension (40 mg/mL). To make the oral suspension, imatinib tablets will be crushed and mixed in Ora-sweet solution to yield a concentration of 40 mg/mL suspension by pharmacy. Additionally, in the absence of supportive microbiological testing results, we confirm that the in-use stability period for the prepared imatinib suspensions will be 24 hours at room temperature or 7 days at refrigerated conditions. The pharmacy staff will follow the American Society Health-System Pharmacists (ASHP) guidelines for handling hazardous drugs.

*Placebo*: The matching placebo will be packaged by Investigational Drug Service Pharmacy at University of Maryland Medical Center. The placebos will be prepared using size 000 capsules and cellulose microcrystalline filler. Imatinib 400 mg capsules and placebo capsules will be identical form and color. For patients on ventilator or ECMO, placebo will be given as oral suspension with similar process for making imatinib suspension.

*Concomitant Medications/supportive care*: In both arms, patients can receive concomitant available local standard of care antipyretics, antibacterials, antivirals, antifungals and anti-inflammatory including hydroxychloroquine at the discretion of the treating physician as necessary. For other drug-drug interactions particularly with CYP P450, the treating physician should consider the risk and benefit of drug administration based on available information.

Co-administration of off-label immunomodulatory treatments for COVID-19 including but not limited to corticosteroids, sarilumab, clazakizumab, tocilizumab, and anakinra will be allowed but may affect interpretability of study outcomes. The timing, dosing, and duration of these treatments will be meticulously collected, including any of these treatments that may be used for participants who experience progression of COVID-19 disease after study enrollment. Two analyses will be performed, the primary analysis will compare the primary endpoint in the two trial arms irrespective of any other treatment; the second analysis will be stratified for co-administration of immunomodulatory drugs.

**Main outcomes:**

The *primary endpoint* is the proportion of patients with a two-point improvement at Day 14 from baseline using the 8-category ordinal scale. The ordinal scale is an evaluation of the clinical status at the first assessment of a given study day. The scale is as follows: 1) Not hospitalized, no limitations on activities; 2) Not hospitalized, limitation on activities and/or requiring home oxygen; 3) Hospitalized, not requiring supplemental oxygen – no longer requires ongoing medical care; 4) Hospitalized, not requiring supplemental oxygen - requiring ongoing medical care (COVID-19 related or otherwise); 5) Hospitalized, requiring supplemental oxygen; 6) Hospitalized, on non-invasive ventilation or high flow oxygen devices; 7) Hospitalized, on invasive mechanical ventilation or ECMO; 8) Death.

The *secondary endpoints* include: All-cause mortality at Day 28, All-cause mortality at Day 60, Time to a 2-point clinical improvement difference over baseline, Duration of hospitalization, Duration of ECMO or invasive mechanical ventilation (for subjects who are on ECMO or mechanical ventilation at Day 1), Duration of ICU stay (for subjects who are in ICU at Day 1), Time to SARS-CoV-2 negative by RT-PCR, Proportion of patients with negative oropharyngeal or nasopharyngeal swab for SARS-CoV-2 by RT-PCR on days 5, 10, 14, 21, and 28 after starting treatment, Proportion of subjects with serious adverse events, Proportion of subjects who discontinue study drug due to adverse events.

The *exploratory endpoints* include: Determine the impact of treatment arms on IL-6 levels, Obtain blood/peripheral blood mononuclear cells (PBMCs) for storage to look at transcriptomics in severe disease, Association of major histocompatibility complex (MHC) with severity of illness, Mean change in the ordinal scale from baseline, Time to an improvement of one category from admission using an ordinal scale, Duration of hospitalization, Duration of new oxygen use, Number of oxygenation free days, Duration of new mechanical ventilation, Number of ventilator free days.

**Randomization:**

Eligible patients will be uniformly randomized in 1:1 ratio to receive either imatinib or placebo for 14 days. Both groups will receive the BCC. The randomized treatment allocations use stratified, permuted block randomization with a variable block size; blocks are generated using a validated random number generator. In order to balance the severity of the respiratory illness between the two arms, randomization will be stratified based on radiographic findings and oxygen requirements: 1) Severe disease: evidence of pneumonia on chest X-ray or CT scan OR chest auscultation (rales, crackles), and SpO_2_ ≤92% on ambient air or PaO_2_/FiO_2_ <300 mmHg, and requires supplemental oxygen administration by nasal cannula, simple face mask, or other similar oxygen delivery device; 2) Critical disease: requires supplemental oxygen delivered by non-rebreather mask or high flow cannula OR use of invasive or non-invasive ventilation OR requiring treatment in an intensive care unit, use of vasopressors, extracorporeal life support, or renal replacement therapy.

**Blinding (masking):**

The participants, caregivers, and the statistician are blinded to group assignment. The only people who are not blinded are Site Pharmacists. Blinding will be performed via a specific randomization process. Centralized, concealed randomization will be executed by the Primary Site’s Pharmacist. Data on eligible consented cases will be submitted electronically on the appropriate on-study form to the pharmacy, where the patient is randomized to imatinib or placebo. Imatinib 400 mg capsules and placebo capsules will be identical form and color. For patients on ventilator or ECMO, placebo will be given as oral suspension with similar process for making imatinib suspension.

**Numbers to be randomized (sample size):**

The trial is designed as a double-blind, two-parallel arm, randomized controlled trial with a uniform (1:1) allocation ratio to: Arm A) Imatinib or Arm B) Placebo. Patients in both arms will receive the BCC per local institutional standards at the discretion of the treating physician.

Group sample sizes of 102 in Arm A and 102 in Arm B achieve 80.6% power to detect a difference between the group proportions of 0.20. The proportion in Arm A (imatinib treatment arm) is assumed to be 0.30 under the null hypothesis and 0.50 under the alternative hypothesis. The proportion in Arm B (placebo control arm) is 0.30. The test statistic used is the two-sided Fisher's Exact Test. The significance level of the test is targeted at 0.05. The significance level actually achieved by this design is α=0.0385. The power of the test is calculated using binomial enumeration of all possible outcomes.

The primary analysis will be conducted using an intention to treat principle (ITT) for participants who at least receive one dose of study drug or placebo. The sample size is not inflated for dropouts. All patients will be evaluable irrespective of the clinical course of their disease.

**Trial Status:**

Current protocol version is 1.2 from May 8, 2020. The recruitment started on June 15, 2020 and is ongoing. We originally anticipated that the trial would finish recruitment by mid 2021. We are aware of the enrollment requirement of approximately 200 patients, which is required to provide scientific integrity of the results. We are also aware of the fact that enrolling this number of patients in a single-site at University of Maryland Medical Center (UMMC) may take longer than expected, particularly taken into account other competing studies. For this reason, we are actively considering opening the protocol in other sites. After identification of other sites, we will fulfill all regulatory requirements before opening the protocol in other sites.

**Trial registration:**

ClinicalTrials.gov Identifier: NCT04394416. First Posted: May 19, 2020; Last Update Posted: June 4, 2020. FDA has issued the “Study May Proceed” Letter for this clinical trial under the Investigational New Drug (IND) number 149239.

**Full protocol:**

The full protocol is attached as an additional file, accessible from the Trials website (Additional file 1). In the interest in expediting dissemination of this material, the familiar formatting has been eliminated; this Letter serves as a summary of the key elements of the full protocol.

**Supplementary information:**

**Supplementary information** accompanies this paper at 10.1186/s13063-020-04819-9.

## Supplementary information

**Additional file 1.**

## Data Availability

The main principal investigator and the corresponding author (AE) and other principal investigators (JVC, RT, JB) will have access to the final trial dataset. The corresponding author will evaluate any request for data sharing and will consult with the other principal investigators after the publication of the main results. Requests can be sent to aemadi@umm.edu.

