# Supplementary material for: Safety and Efficacy of Imatinib for Hospitalized Adults with COVID-19: A structured summary of a study protocol for a randomised controlled trial
Source: Trials. 2020 Oct 28;21:897. doi: 10.1186/s13063-020-04819-9 (PMC7594416; doi:10.1186/s13063-020-04819-9)
Supplement: Supplementary file 1 — Additional file 1. [file 13063_2020_4819_MOESM1_ESM.pdf]

# Randomized Double-Blind Placebo-Controlled Trial on the Safety and Efficacy of Imatinib for Hospitalized Adults with COVID-19

## Investigational Agent: Imatinib

**Protocol Number: 2038GCCC**
**HRPO Number: HP-00090890**
**IND # 149239**
**NCT # *****


## Sponsor:

**Ashkan Emadi, M.D., Ph.D.**
**University of Maryland Greenebaum Comprehensive Cancer Center**
22 South Greene Street
Baltimore, MD 21201 USA


**Protocol Version: 1.2**

**May 8, 2020**


CONFIDENTIAL

The information contained in this protocol is confidential and is intended for the use of the clinical investigators and their study staff. The information in this document is the property of University of Maryland Baltimore, and may not be disclosed unless federal or state law or regulations require such disclosure. This information may be disclosed only to those persons involved in the study who have a need to know, with the obligation not to further disseminate this information.

# Key Personnel

**Principal Investigator:**
Ashkan Emadi, M.D., Ph.D.
Professor of Medicine and Pharmacology
University of Maryland, School of Medicine
22 South Greene St., Room N9E06
Baltimore, MD 21201
Telephone: 410-328-2596
Fax: 410-328-6896
Email: aemadi@umm.edu

**Co-Investigator:**
John Baddley, M.D.
Professor of Medicine
University of Maryland School of Medicine, Institute of Human Virology
725 W. Lombard Street, Room S516
Baltimore, MD 21201
Telephone: 410-706-6444
Fax: 410-706-1992
Email: jbaddley@ihv.umaryland.edu

Joel V. Chua, M.D.
Assistant Professor of Medicine
Chief, Surgical Infectious Disease, UMMC
University of Maryland School of Medicine, Institute of Human Virology
725 West Lombard Street, Room N146
Baltimore, MD 21201
Telephone: 410-706-5704
Fax: 410-706-3243
Email: jchua@ihv.umaryland.edu

Rohit Talwani, M.D.
Associate Professor of Medicine
University of Maryland School of Medicine, Institute of Human Virology
725 West Lombard Street
Baltimore, MD 21201
Telephone: 410-706-8264
Fax: 410-706-4619
E-mail: rtalwani@ihv.umaryland.edu

**Statistician:** Søren Bentzen, Ph.D., D.Sc.

**Lead Protocol Coordinator**: Monica Estrada, Email: Monica.estrada@umm.edu

**Lead Data Manager:** Kirsten Antonin, Email: Kirsten.Antonen@umm.edu

# Protocol Synopsis

**Title:** Randomized Double-Blind, Placebo-Controlled Trial on the Safety and Efficacy of Imatinib for Hospitalized Adults with COVID-19.

**Study Phase:** 3

**Background and Rationale:**
Coronavirus disease 2019 (COVID-19) is an ongoing global pandemic caused by the severe acute respiratory syndrome coronavirus 2 (SARS-CoV-2), and at present with no approved or proven antiviral treatment.

Imatinib is a tyrosine kinase inhibitor that has been approved for treatment of many hematologic and solid neoplasm. Imatinib is a weak base that compared to the extracellular compartment is enriched over 1000-fold in the lysosome within several hours as a result of its lysosomotropic property. Imatinib as a weak base accumulates in lysosomes resulting in some antiviral activities by lysosomal alkalization required for virus/cell fusion.

Imatinib demonstrates *in vitro* activity against SARS-CoV viruses. Imatinib inhibit SARS-CoV and MERS-CoV with micromolar $EC_{50}$s (range, 9.8 to 17.6 µM) with low toxicity. The mechanism of action studies suggested that ABL-1 tyrosine kinase regulates budding or release of poxviruses and Ebola virus, demonstrating that the c-ABL-1 kinase signaling pathways play an important role in the egress of these viruses. It is also reported that kinase signaling may also be important for replication of two members of the Coronaviridae family, SARS-CoV and MERS-CoV. *In vivo* studies performed in the mouse model of vaccinia virus infection showed that imatinib was effective in blocking dissemination of the virus.

Imatinib has anti-inflammatory activity including its effectiveness in a "two-hit" murine model of acute lung injury (ALI) caused by combined lipopolysaccharide (LPS) and ventilator-induced lung injury (VILI). Imatinib significantly decreased bronchoalveolar lavage protein, total cells, neutrophils, and TNFα levels in mice exposed to LPS plus VILI, indicating that it attenuates ALI in this clinically relevant model. In another experiment, imatinib attenuated ALI when given 4 hours after LPS, suggesting potential efficacy when given after the onset of injury. Overall, these results strongly suggest the therapeutic potential of imatinib against inflammatory vascular leak and a potential role of imatinib combination therapy for patients with acute respiratory distress syndrome (ARDS) on mechanical ventilation.

We *hypothesize* that addition of imatinib to the best conventional care (BCC) improves the outcome of hospitalized adult patients with COVID-19. This hypothesis is on the bases of 1) intralysosomal entrapment of imatinib will increase endosomal pH and effectively decrease SARS-CoV-2/cell fusion, 2) kinase inhibitory activity of imatinib will interfere with budding/release or replication of SARS-CoV-2, and 3) because of the critical role of mechanical ventilation in the care of patients with ARDS, imatinib will have a significant clinical impact for patients with severe COVID-19 infection in Intensive Care Unit (ICU).

**Target Population:**
Hospitalized adult patients with COVID-19.

**Hypothesis:**
Addition of imatinib to the BCC will provide a superior clinical outcome for patients with COVID-19 compared with BCC plus placebo.

**Primary Objectives**
To evaluate the efficacy, safety, and tolerability of combination of imatinib compared with placebo in combination with the BCC in adults hospitalized with COVID-19.

**Primary Endpoint**
The primary endpoint is proportion of patients with a two-point improvement at Day 14 from baseline using the 8-category ordinal scale.

The ordinal scale is an evaluation of the clinical status at the first assessment of a given study day. The scale is as follows: 1) Not hospitalized, no limitations on activities; 2) Not hospitalized, limitation on activities and/or requiring home oxygen; 3) Hospitalized, not requiring supplemental oxygen – no longer requires ongoing medical care; 4) Hospitalized, not requiring supplemental oxygen - requiring ongoing medical care (COVID-19 related or otherwise); 5) Hospitalized, requiring supplemental oxygen; 6) Hospitalized, on non-invasive ventilation or high flow oxygen devices; 7) Hospitalized, on invasive mechanical ventilation or extracorporeal membrane oxygenation (ECMO); 8) Death.

**Secondary Endpoints**

- All-cause mortality at Day 28
- All-cause mortality at Day 60
- Time to a 2-point clinical improvement difference over baseline
- Duration of hospitalization
- Duration of ECMO or invasive mechanical ventilation (for subjects who are on ECMO or mechanical ventilation at Day 1)
- Duration of ICU stay (for subjects who are in ICU at Day 1)
- Time to SARS-CoV-2 negative by reverse transcriptase-polymerase chain reaction (RT-PCR)
- Proportion of patients with negative oropharyngeal or nasopharyngeal swab for SARS-CoV-2 by quantitative RT PCR on days 5, 10, 14, 21, and 28 after starting treatment.
- Proportion of subjects with serious adverse events
- Proportion of subjects who discontinue study drug due to adverse events

**Study design**

- This is an individual patient-level randomized, double-blind, placebo-controlled phase 3 study to evaluate the safety and efficacy of imatinib for the treatment of hospitalized adults with COVID-19. Participants will be followed for up to 60 days from start of study drug administration. Eligible patients will be randomized in 1:1 ratio to receive either imatinib or placebo for 14 days. Both groups will receive the best conventional care (BCC, detailed in Section 1.6).
- In order to balance the severity of the respiratory illness between the two arms, per FDA recommendation randomization will be stratified based on radiographic findings and oxygen requirements:
  1) **Severe disease:** evidence of pneumonia on chest X-ray or CT scan OR chest auscultation (rales, crackles), and $SpO_2 \leq 92\%$ on ambient air or $PaO_2/FiO_2 < 300$ mmHg, and requires supplemental oxygen administration by nasal cannula, simple face mask, or other similar oxygen delivery device;
  2) **Critical disease:** requires supplemental oxygen delivered by non-rebreather mask or high flow cannula OR use of invasive or non-invasive ventilation OR requiring

treatment in an intensive care unit, use of vasopressors, extracorporeal life support, or renal replacement therapy.

- This trial will be conducted in accordance with the principles of the Declaration of Helsinki and the Good Clinical Practice guidelines of the International Conference on Harmonization.

**Sample Size:**

The trial is designed as a double-blind, two-parallel arm, randomized controlled trial with a uniform (1:1) allocation ratio to: Arm A) Imatinib or Arm B) Placebo. Patients in both arms will receive the best conventional care (BCC) per local institutional standards at the discretion of the treating physician.

Group sample sizes of 102 in Arm A and 102 in Arm B achieve 80.6% power to detect a difference between the group proportions of 0.20. The proportion in Arm A (imatinib treatment arm) is assumed to be 0.30 under the null hypothesis and 0.50 under the alternative hypothesis. The proportion in Arm B (placebo control arm) is 0.30. The test statistic used is the two-sided Fisher's Exact Test. The significance level of the test is targeted at 0.05. The significance level actually achieved by this design is $\alpha=0.0385$. The power of the test is calculated using binomial enumeration of all possible outcomes.

The primary analysis will be conducted using an intention to treat principle (ITT) for participants who at least receive one dose of study drug or placebo. The sample size is not inflated for dropouts. All patients will be evaluable irrespective of the clinical course of their disease.

**Key Eligibility Criteria**

*Inclusion Criteria*

Patients may be included in the study only if they meet **all** of the following criteria:

- Ability to understand and willingness to sign a written informed consent document. Informed consent must be obtained prior to participation in the study. For patients who are too unwell to provide consent such as patients on invasive ventilator or ECMO, Legally Authorized Representative (LAR) can sign the informed consent.
- Hospitalized patients ≥ 18 years of age
- Positive RT-PCR assay for SARS-CoV-2 in the respiratory tract sample (oropharyngeal, nasopharyngeal or BAL) by Center for Disease Control or local laboratory within 7 days of randomization.

*Exclusion Criteria*

Patients meeting any of the following criteria are **not** eligible for the study:

- Patients receiving any other investigational agents in a clinical trial. Off-label use of agents such as hydroxychloroquine is not an exclusion criterion.
- Pregnant or breastfeeding women.
- Patients with significant liver or renal dysfunction function at screen as defined as:
    - Direct bilirubin > 2.5 mg/dL
    - AST, ALT, or alkaline phosphatase > 5 x upper limit of normal
    - eGFR ≤ 30 mL/min or requiring renal replacement therapy
- Patients with significant hematologic disorder at screen as defined as:
    - Absolute neutrophil count (ANC) < 500/µL
    - Platelet < 20,000/µL
    - Hemoglobin < 7 g/dL

- Uncontrolled undercurrent illness including, but not limited to, symptomatic congestive heart failure, unstable angina pectoris, uncontrolled active seizure disorder, or psychiatric illness/social situations that per site Principal Investigator's judgment would limit compliance with study requirements.
- Known allergy to imatinib or its component products.
- Any other clinical conditions that in the opinion of the investigator would make the subject unsuitable for the study.

**Study Treatments**: Imatinib oral 400 mg daily or placebo for 14 days.

**Study Centers:** University of Maryland Medical Center, Baltimore, MD as the initiating site. The study may be opened in other centers including University of Maryland Medical System hospitals and other centers across the United States on the bases of the accrual rate or the magnitude of COVID-19 pandemic. With the current number of COVID-19 in the US, the status in Maryland is still uncertain. (See: COVID DATA VISUALIZATION LINKS: IHME Data Visualization: select Maryland, https://covid19.healthdata.org/projections). We are aware of the enrollment requirement of ~200 patients, which is required to provide scientific integrity of the results. We are also aware of the fact that enrolling this number of patients in a single-site at UMMC may take longer than expected particularly taken into account other competing studies. For this reason, we actively consider to open the protocol in other sites. After identification of other sites, we will fulfill all regulatory requirements before opening the protocol in other sites.

# Schedule of Activities

| ASSESSMENTS/ OBSERVATIONS | Screen (D-3 to D1) | Day 1 | Day 2 | Day 5 | Day 8 | Day 10 | Day 14[a] | Day 21 | Day 28 | Day 60[b] |
|---|---|---|---|---|---|---|---|---|---|---|
| | | | | | | | ±2 d | ±2 d | ±3 d | ±7 d |
| | | | | | | | | | | |
| Informed consent | X | | | | | | | | | |
| Confirm eligibility | X | X | | | | | | | | |
| Randomization | | X | | | | | | | | |
| Study drug administration[c] | | X | | | | | | | | |
| | | | | | | | | | | |
| Demographics | X | | | | | | | | | |
| Medical history | X | | | | | | | | | |
| Vital signs & Physical exam[d] | X | X | | X | | X | X | X | X | X |
| Clinical assessments[e] | X | X | | | | | | | | |
| CBC w diff count | X | | X | X | | X | X | | X | X |
| Chemistries[f] | X | | X | X | | X | X | | X | X |
| CRP | X | | | X | | | X | | X | X |
| ECG | X | | | | | | | | | |
| Pregnancy test | X | | | | | | | | | |
| AE assessment | | X | | | | | | | | |
| Concomitant medication | X | X | | X | | X | X | X | X | X |
| Primary endpoint assessment | | | | | | | X | | | |
| SARS-CoV-2 PCR[g] | | X | | X | | X | X | | X | |
| Correlative/scientific studies | | X | | X | | X | X | | X | X |

Screen (Day -3 to Day 1) - Screening and Day 1 visits can occur the same day.

[a] Early discharge visit - use Day 14 assessments
[b] Early termination visit - use Day 60 assessments
[c] For planned 14 days

[d] Clinically appropriate physical exams including vital signs and $O_2$ saturation

[e] Clinical assessments - 7-point ordinal scale and respiratory treatment status. Daily while hospitalized.
[f] Chemistries include the following: sodium, potassium, chloride, bicarbonate, BUN, creatinine, glucose, calcium, magnesium, phosphorous, AST, ALT, total bilirubin, alkaline phosphatase, albumin, total protein, and LDH. If total bilirubin is elevated, then fractionated direct bilirubin can be measured. Amylase, lipase, coagulation factors, and uric acid are measured at the discretion of the investigators. Check D-dimer and ferritin levels when clinically indicated.

[g] Respiratory specimens (nasopharyngeal or oropharyngeal swabs)

## Study Glossary

| | |
|---|---|
| AE | Adverse Event |
| ALP | Alkaline Phosphatase |
| ALT | Alanine Aminotransferase |
| ANC | Absolute Neutrophil Count |
| AST | Aspartate Aminotransferase |
| BCC | Best Conventional Care |
| BP | Blood Pressure |
| CBC | Complete Blood Count |
| CFR | The Code of Federal Regulations |
| CI | Confidence Interval |
| CNS | Central Nervous System |
| CRF | Case Report Form |
| CRP | C Reactive Protein |
| CRS | Cytokine Release Syndrome |
| CTCAE | Common Terminology Criteria for Adverse Events |
| DOR | Duration Of Response |
| DSM | Data and Safety Monitoring |
| ECG | Electrocardiogram |
| eCRF | Electronic Case Report Form |
| FDA | Food and Drug Administration |
| FDAAA | Food and Drug Administration Amendments Act |
| FDAMA | Food and Drug Administration Modernization Act |
| GCP | Guideline for Good Clinical Practice |
| h, hr | Hour |
| Hgb | Hemoglobin |
| HR | Heart Rate |
| ICF | Informed Consent Form |
| ICH | International Conference on Harmonization |
| ICJME | International Committee of Medical Journal Editors |
| IND | Investigational New Drug |
| IPIM | Investigational Product Instructional Manual |
| IRB | Institutional Review Board |
| IV | Intravenous |
| LDH | Lactate Dehydrogenase |
| MHC | Major Histocompatibility Complex |
| mL | microliter |
| mg | Milligrams |
| MOI | Multiplicity Of Infection |
| N, n | Number |
| OS | Overall Survival |
| PBMC | Peripheral Blood Mononuclear Cells |
| PK | Pharmacokinetic |
| PCR | Polymerase Chain Reaction |
| QAC | Quality Assurance Committee |
| RT-PCR | Reverse-Transcriptase Polymerase Chain Reaction |
| RBC | Red Blood Cell |
| SAE | Serious Adverse Event |
| SD | Standard Deviation |
| SEM | Standard Error of Mean |
| SFU | Safety Follow-UP |
| SOC | Standard Of Care |
| TBL | Total Bilirubin |
| VPN | Virtual Protected Network |
| WBC | White Blood Cell |
| WHO | World Health Organization |

# Table of Contents


Key Personnel ........ 2

Protocol Synopsis ........ 3

Schedule of Activities ........ 7

Study Glossary ........ 8

1. BACKGROUND, CONCEPT AND RATIONAL ........ 11
   - 1.1. COVID-19 ........ 11
   - 1.2. Imatinib and lysosomal Sequestration ........ 11
   - 1.3. Anti-Viral Activity of Imatinib ........ 12
   - 1.4. Anti-Inflammatory Activity of Imatinib ........ 13
   - 1.5. Rationale: Imatinib for Treatment of COVID-19 ........ 16
   - 1.6. Best Conventional Care ........ 19

2. OBJECTIVES AND ENDPOINTS ........ 20
   - 2.1. Primary Objectives ........ 20
   - 2.2. Primary Endpoint ........ 20
   - 2.3. Secondary Endpoints ........ 20
   - 2.4. Exploratory Endpoints ........ 20

3. CRITERIA FOR STUDY SUBJECT ELIGIBILITY ........ 21
   - 3.1. Inclusion Criteria ........ 21
   - 3.2. Exclusion Criteria ........ 21
   - 3.3. Inclusion of Women and Minorities ........ 21

4. RECRUITMENT PLAN AND REGISTRATION PROCEDURES ........ 22

5. EXPERIMENTAL PLAN ........ 23
   - 5.1. Study Design ........ 23
   - 5.2. Study Drugs ........ 24
   - 5.3. Replacement of Subject ........ 25
   - 5.4. Estimated Study Duration and End of Study ........ 25

6. DOSE MODIFICATION AND STUDY DISCONTINUATION ........ 25
   - 6.1. Dose Modification and Interruption ........ 25
   - 6.2. Study Discontinuation ........ 25

7. STUDY ASSESSMENTS/PROCEDURES ........ 26
   - 7.1. Informed Consent and Demographics ........ 26
   - 7.2. Medical History ........ 27
   - 7.3. General Physical Examination ........ 27
   - 7.4. Laboratory Assessments ........ 27
   - 7.5. Schedule of Assessments (Study Calendar) ........ 28

8. REPORTING ADVERSE EVENTS / REGULATORY REQUIREMENTS ........ 29

8.1. Definitions ... 29
8.2. Reporting of Adverse Events ... 29
8.3. Reporting of Serious Adverse Events ... 30
8.4. Data Safety Monitoring Plan ... 30
8.5. Compliance with Laws and Guidance ... 31
8.6. Institutional Review Board (IRB) ... 31
8.7. Clinical Trial Monitoring ... 31
8.8. FDA Reporting ... 31
8.9. Records Retention ... 32
8.10. Compliance with Trial Registration and Results Posting Requirements ... 32
8.11. Data Management ... 32

9. ADMINISTRATIVE AND LEGAL OBLIGATION ... 32
9.1. Protocol Amendments and Study Termination ... 32
9.2. Investigator Responsibilities for Data Collection ... 33
9.3. Language ... 33
9.4. Publication Policy ... 33
9.5. Compensation ... 33

10. STATISTICAL CONSIDERATIONS ... 34
10.1. Randomization ... 34
10.2. Primary endpoint ... 34
10.3. Sample size estimation and power ... 34
10.4. Secondary endpoints ... 34
10.5. Interim analysis ... 35
10.6. Safety profile ... 35
10.7. Analysis of Efficacy Endpoints ... 35

11. CORRELATIVE SCIENTIFIC STUDIES ... 35
11.1. Viral Shedding ... 35
11.2. Peripheral blood collection ... 35
11.3. Serology ... 35

12. REFERENCES ... 36

# 1. BACKGROUND, CONCEPT AND RATIONAL

## 1.1. COVID-19

A novel coronavirus, called SARS-CoV-2, has caused a pandemic of respiratory illness termed COVID-19.[1,2] By April 24th 2020, approximately 2,800,000 confirmed cases are reported from 187 countries, areas or territories with approximately 194,000 confirmed deaths.[3] In the US, there are ~900,000 confirmed cases with ~50,000 deaths.[3] The clinical manifestation of COVID-19 ranges from self-limiting and mild respiratory tract illness in approximately 80% of cases to progressive pneumonia, multi-organ failure, and death.[4,5] Although several groups around the globe are actively working on development of treatment(s) for COVID-19; to date, no specific small molecule, biologics including monoclonal antibodies, or vaccine has been approved for this viral disease.

## 1.2. Imatinib and lysosomal Sequestration

Imatinib mesylate is a small molecule kinase inhibitor approved by the FDA for the treatment of patients with Philadelphia chromosome positive (Ph+) chronic myeloid leukemia (CML), Ph+ acute lymphoblastic leukemia (Ph+ ALL), myelodysplastic/myeloproliferative diseases (MDS/MPD) associated with PDGFR (platelet-derived growth factor receptor), systemic mastocytosis (SM) without the D816V c-Kit mutation, hypereosinophilic syndrome (HES) and/or chronic eosinophilic leukemia (CEL) who have the FIP1L1-PDGFRα fusion kinase, unresectable, recurrent and/or metastatic dermatofibrosarcoma protuberans, Kit (CD117) positive unresectable and/or metastatic as well as adjuvant treatment of malignant gastrointestinal stromal tumors (GIST).

Imatinib film-coated tablets contain imatinib mesylate equivalent to 100 mg or 400 mg of imatinib free base. Imatinib mesylate is designated chemically as 4-[(4-Methyl-1-piperazinyl)methyl]-N-[4-methyl-3-[[4-(3-pyridinyl)-2-pyrimidinyl]amino]-phenyl]benzamide methanesulfonate and its structural formula is:

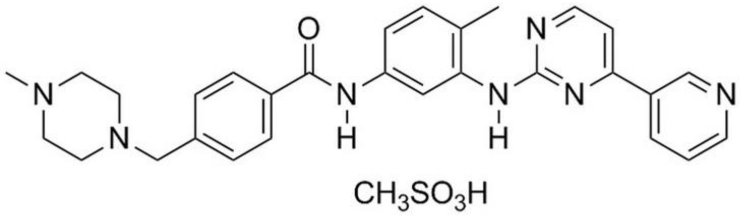


Imatinib mesylate is soluble in aqueous buffers ≤ pH 5.5 but is slightly soluble to insoluble in neutral/alkaline aqueous buffers. Lysosomal sequestration and accumulation of weak base tyrosine kinase inhibitors (TKIs) including imatinib has been reported by several groups.[6-9]

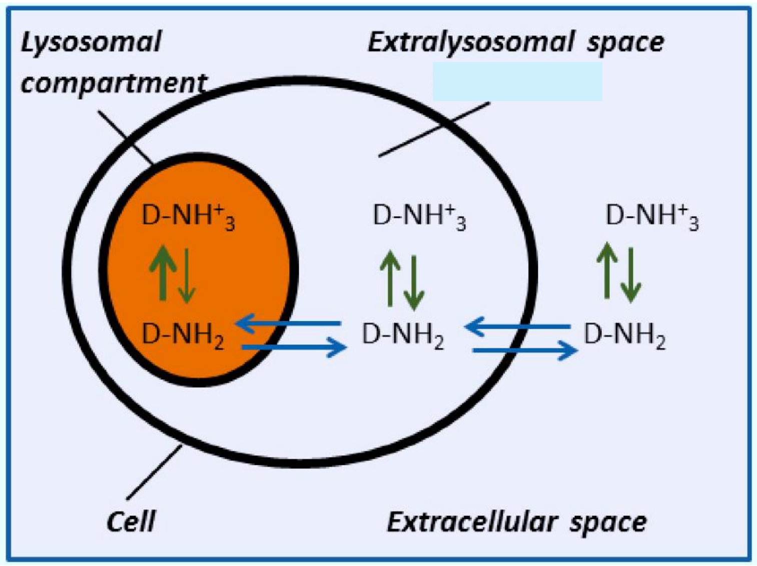


**Figure 1.** Weak-base drug distribution with lysosomal sequestration, uncharged molecules can freely diffuse across the lysosomal and plasma membranes while protonated (charged) molecules are trapped inside the acidic environment of lysosomes[10]

In a study using fluorescent microscopy to establish subcellular localization of imatinib, the investigators reported high intracellular uptake and retention (IUR) of imatinib in different cell lines.[11] The IUR of imatinib was time-, dose-, temperature-, and energy-dependent suggesting that plasma membrane imatinib transporters do not substantially contribute to the IUR of imatinib. Importantly, co-exposure with weak bases such as prazosin, amantadine, and $NH_4Cl$ significantly altered the IUR of imatinib, likely due to lysosomal retention and accumulation of imatinib. Co-staining experiments with LysoTracker Red confirmed lysosomal sequestration of imatinib. The authors concluded that intracellular imatinib levels are primarily determined by lysosomal sequestration of imatinib and do not depend on the expression of imatinib transporters on plasma membrane.[11]

In another study, the investigator reported the direct label-free visualization and quantification of imatinib inside living cells using hyperspectral stimulated Raman scattering imaging.[12] Compared to the extracellular drug, imatinib was enriched over 1000-fold in the lysosome within several hours as a result of its lysosomotropic property. Co-administration of imatinib with high dose chloroquine alkalinized intralysosomal vesicle, slightly (~5-fold) decreased intralysosomal concentration of imatinib but did not change the intralysosomal concentration of chloroquine.[12] This study confirmed the accumulation and intracellular drug-drug interaction of weak bases such as imatinib and chloroquine with label-free imaging.

## 1.3. Anti-Viral Activity of Imatinib

Imatinib mesylate is a known inhibitor of the Abelson murine leukemia viral oncogene homolog 1 (ABL1) pathway. The ABL1 pathway is a signaling pathway involved in cell differentiation, cell adhesion, and the cellular stress response. Overactivation of the ABL1 pathway can lead to CML. Imatinib mesylate inhibit SARS-CoV and MERS-CoV with micromolar $EC_{50}$s (range, 9.8 to 17.6 µM) and low toxicity (Figure 2).[13] SARS-CoV does appear to be more sensitive to the ABL1 inhibitor than MERS-CoV.

Imatinib mesylate has been reported to block egress of Ebola virus and of poxviruses and entry of coxsackievirus.[14-16] These data suggest that the ABL1 pathway may be important for replication of many different virus families and, therefore, inhibitors of this pathway have the

potential to be broad-spectrum antivirals.[13] Mechanism of action studies showed that ABL1 tyrosine kinase controls budding or release of poxviruses and Ebola virus, demonstrating that the c-ABL1 kinase signaling pathways may play a role in the egress of these viruses. Frieman and his colleagues reported that "kinase signaling may also be important for replication of two members of the Coronaviridae family, SARS-CoV and MERS-CoV. Imatinib mesylate."[13] The step in viral replication in which these kinases are involved will need to be investigated further. *In vivo* studies performed in the mouse model of vaccinia virus infection demonstrated that imatinib mesylate was effective in blocking dissemination of the virus.[16]

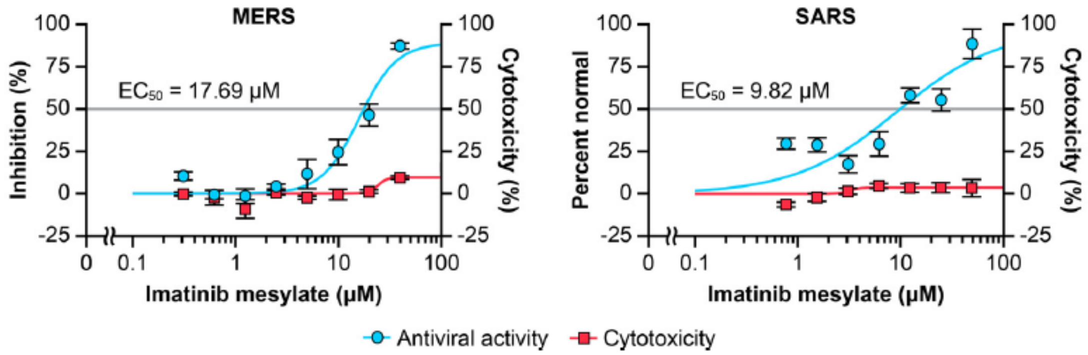


**Figure 2.** Antiviral activity of imatinib mesylate. Vero E6 cells were infected with MERS-CoV or SARS-CoV at an MOI of 0.1 or 1, respectively, and treated for 48 h with eight doses of imatinib mesylate (B). Antiviral activity is shown in blue, and cytotoxicity is shown in red. $EC_{50}$s are indicated. Results are representative of one experiment (means SEM; n = 2).[13]

## 1.4. Anti-Inflammatory Activity of Imatinib

Anti-inflammatory activity of imatinib has been reported in preclinical and clinical settings. Amelioration of bleomycin-induced[17] and radiation-induced[18] pulmonary inflammation and fibrosis in mouse models were reported by imatinib as monotherapy or in combination with other agents. Wolf and colleagues provided strong evidence that imatinib has potent anti-inflammatory effects by reporting that it potently inhibited LPS- and Con A-induced TNFα production by human myeloid cells *in vitro* (peripheral blood mononuclear cells, CD14-selected monocytes, and monocyte-derived macrophages).[19] Using several murine models of acute hepatitis, the investigator verified the *in vitro* findings, as imatinib prevented macrophage- and TNFα-dependent inflammatory damage of the liver induced by injection of either galactosamine or LPS by inhibition of hepatic TNFα production.[19]

Interestingly, and perhaps the most relevant to COVID-19-induced acute lung injury/acute respiratory distress syndrome (ALI/ARDS) is the study performed by Rizzo and colleagues.[20] The investigators assessed the effectiveness of imatinib in a "two-hit" model of ALI caused by combined LPS and ventilator-induced lung injury (VILI), See Figures 2 and 3. Imatinib significantly decreased bronchoalveolar lavage protein, total cells, neutrophils, and TNFα levels in mice exposed to LPS plus VILI, indicating that it attenuates ALI in this clinically relevant model, See Figure 4.[20] In another experiment, imatinib attenuated ALI when given 4 hours after LPS, suggesting potential efficacy when given after the onset of injury.[20] Overall, these results strongly suggest the therapeutic potential of imatinib against inflammatory vascular leak and a potential role of imatinib combination therapy for patients with ARDS on mechanical ventilation.

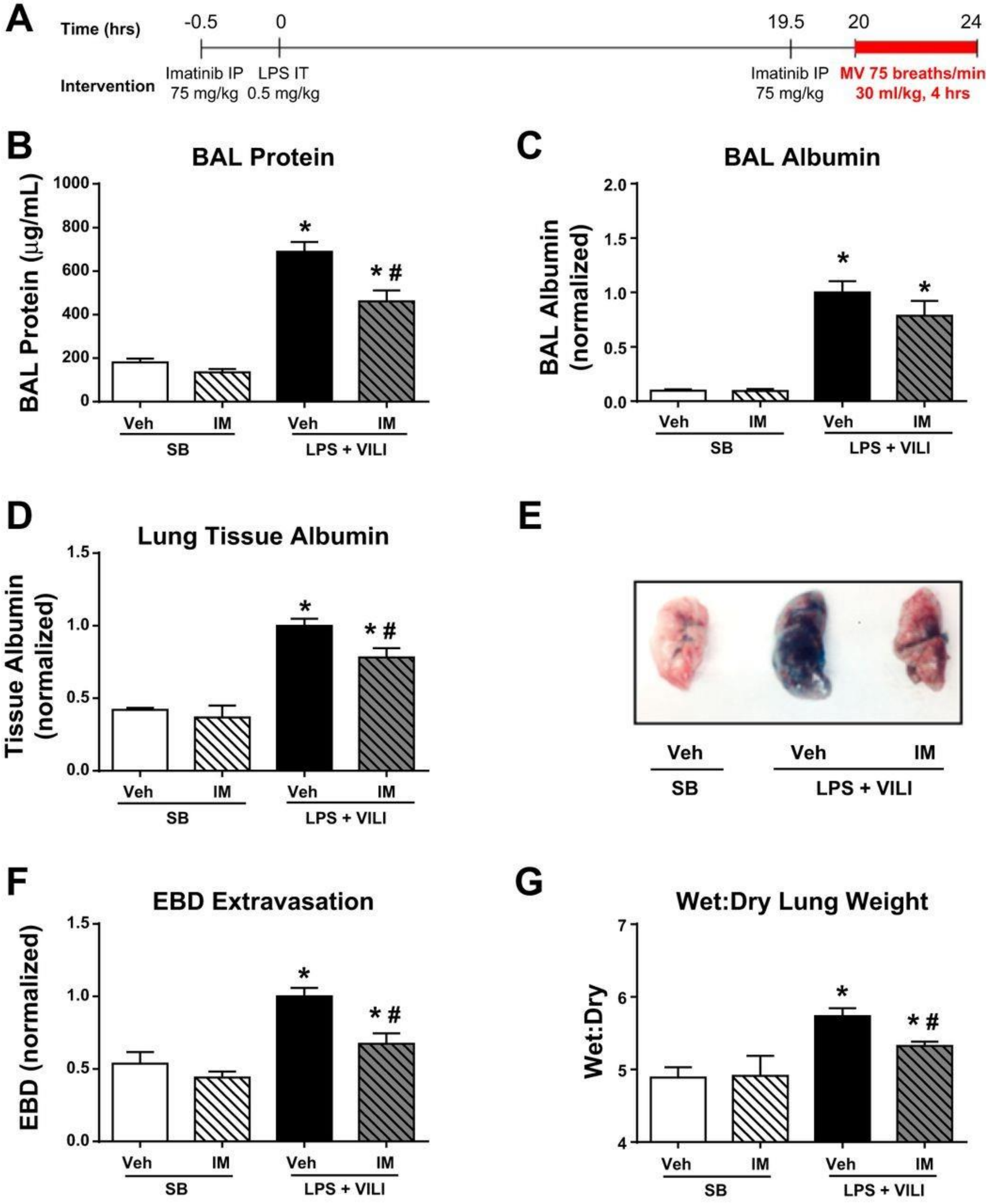


**Figure 3.** Imatinib attenuates vascular leak in mice challenged with a 2-hit model of LPS and mechanical ventilation (MV). Mice were challenged with LPS [0.5 mg/kg, intratracheally (it)] (*t* = 0 h) and MV (respiratory rate 75, tidal volume 30 ml/kg, positive end-expiratory pressure 0 cm $H_2O$) (*t* = 20–24 h) as outlined in *A*. Spontaneously breathing (SB) control mice received PBS (vs. LPS) and were intubated for 4 h without MV. Bronchoalveolar lavage (BAL) fluid, plasma, and lungs were harvested from the animals immediately after MV. Imatinib (IM) (75 mg/kg, ip) or vehicle was administered 0.5 h before LPS administration and before the initiation of MV. Lung permeability was assessed by BAL protein content (*B*), BAL fluid albumin (*C*), and lung tissue albumin (*D*). Additionally, in separate animals, Evans Blue dye (EBD) was injected (30 mg/kg, iv) 1 h before harvest, and representative extravasation into harvested lung tissue is shown (*E*) and quantified in multiple samples (*F*). The left lung of each of these animals was used for calculation of lung wet:dry ratio (*G*). SB ($n = 3$), SB + imatinib ($n = 3$), LPS + ventilator-induced lung injury (VILI) ($n$ = 3–11) and LPS + VILI + imatinib ($n$ = 3–6). *$P < 0.05$ compared with SB controls and #$P < 0.05$ compared with untreated animals.[20]

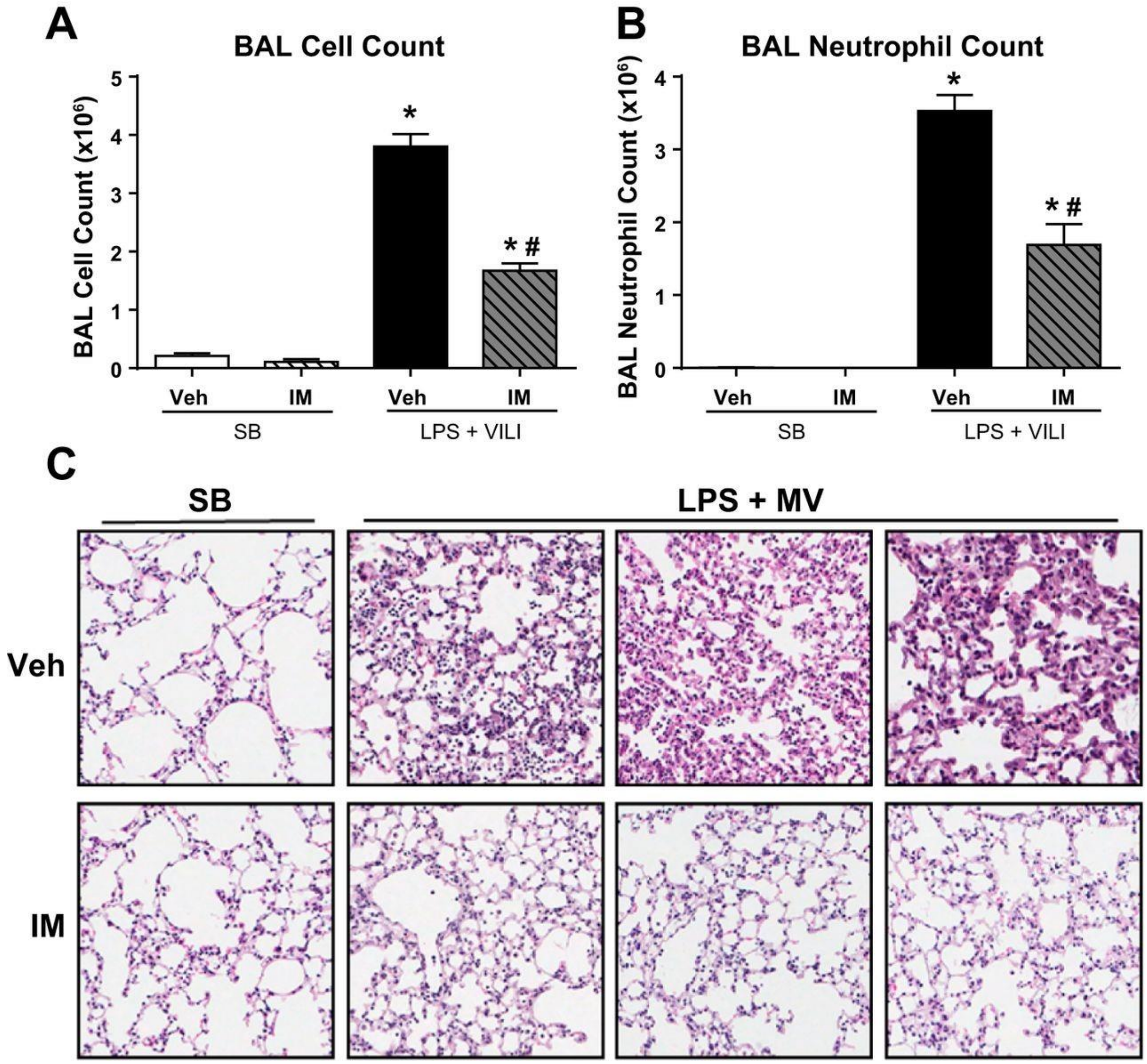


**Figure 4.** Imatinib decreases lung inflammation in mice challenged with LPS and MV. Mice were subjected to the 2-hit lung injury model (LPS + VILI), and lung inflammation was quantified by BAL total cell counts (A) and BAL neutrophil counts (B). Representative hematoxylin and eosin (H and E)-stained lung sections are shown (C). Each H and E image was obtained from a different animal. SB (n = 3), SB + imatinib (n = 3), LPS + VILI (n = 3–11), and LPS + VILI + imatinib (n = 3–6). *P < 0.05 compared with SB controls and #P < 0.05 compared with untreated animals.[20]

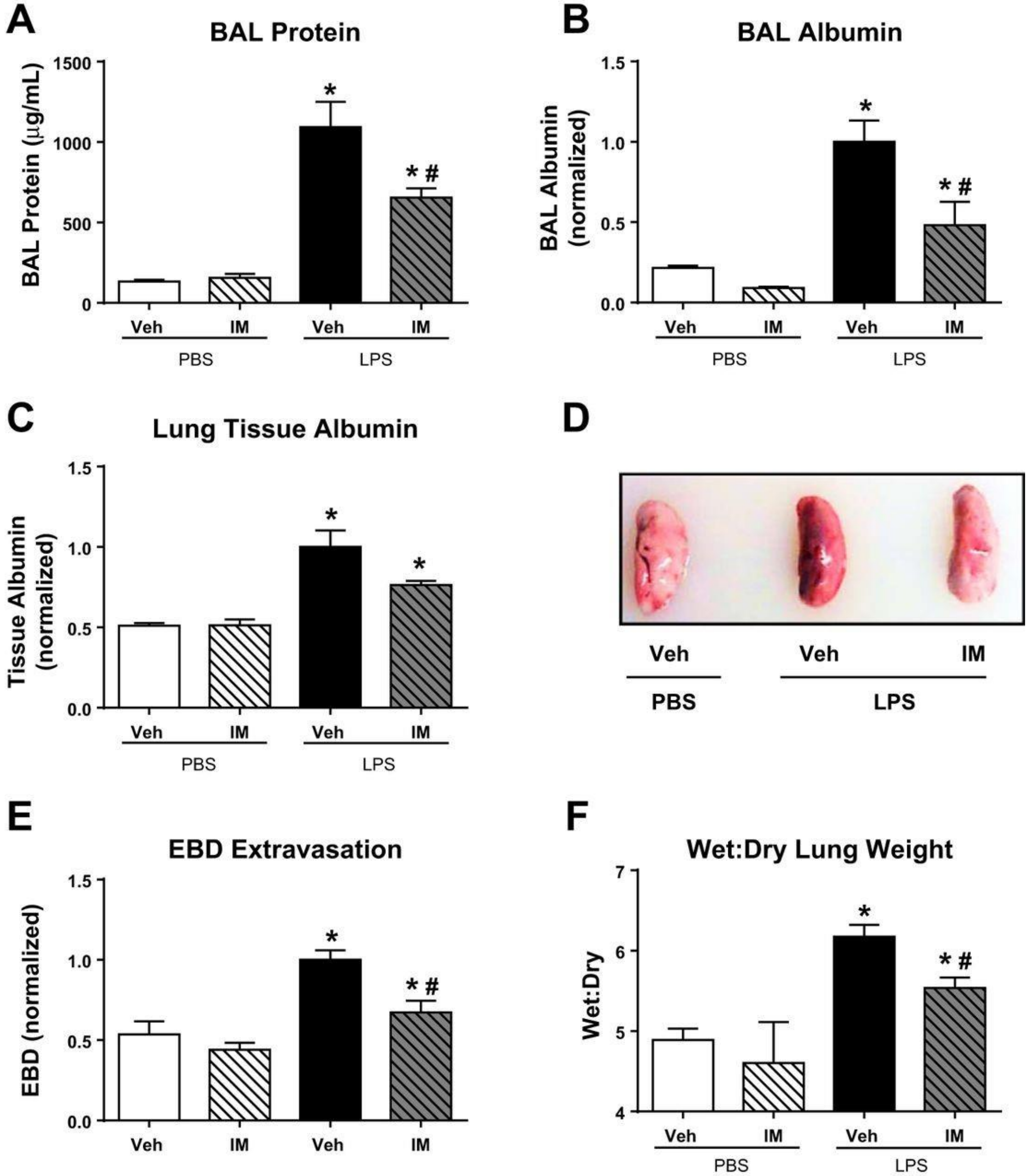


**Figure 5.** Imatinib decreases LPS-induced vascular leak when given after injury onset. Mice were challenged with LPS (1.0 mg/kg, it) (vs. PBS) and then received imatinib (75 mg/kg, ip) (vs. vehicle) 4 h later. Samples were harvested 18 h after LPS administration. Pulmonary vascular permeability was quantified in these animals by measuring BAL protein (A), BAL albumin (B), and lung tissue albumin (C). In separate animals, EBD was injected (30 mg/kg, iv) 1 h before harvest, and representative extravasation into harvested lung tissue is shown (D) and quantified in multiple samples (E). The left lung of each of these animals was used for calculation of lung wet:dry ratio (F). SB (n = 3), SB + imatinib (n = 3), LPS (n = 3–6), and LPS + imatinib (n = 3–6). *P < 0.05 compared with SB controls and #P < 0.05 compared with untreated animals.[20]

## 1.5. Rationale: Imatinib for Treatment of COVID-19

Tolerable and efficacious treatment for patients with COVID-19 are a rapidly evolving therapeutic challenge and the need to develop novel and optimal regimens to treat infection or prevent progression to critical illness remains probably the most important medical challenge in early 2020 worldwide. There are numerous clinical trials currently ongoing across the globe

using/repurposing anti-virals, anti-malaria agents, cytokines, monoclonal antibodies and vaccines. In this protocol, and based on preclinical evidence, we aim to clinically examine the addition of imatinib to BCC for treatment of hospitalized patients with COVID-19 positivity.

Imatinib is a weak base that accumulate in lysosomes resulting in potential broad-spectrum antiviral activities by lysosomal alkalization required for virus/cell fusion of SARS-CoV.[21] It is demonstrated that imatinib can be trapped intralysosomally and increase endosomal pH[12] necessary for SARS-CoV-2 cell/membrane fusion and replication. Moreover, imatinib has anti-inflammatory effect in acute lung injury. Because of the critical role of mechanical ventilation in the care of patients with ARDS, this can have a significant clinical impact for patients with severe COVID-19 infection in ICU.[20] Mechanistic studies in mouse lung tissue and human lung endothelial cells revealed that imatinib inhibits LPS-induced NF-κB expression and activation, Figures 5 and 6.[20]

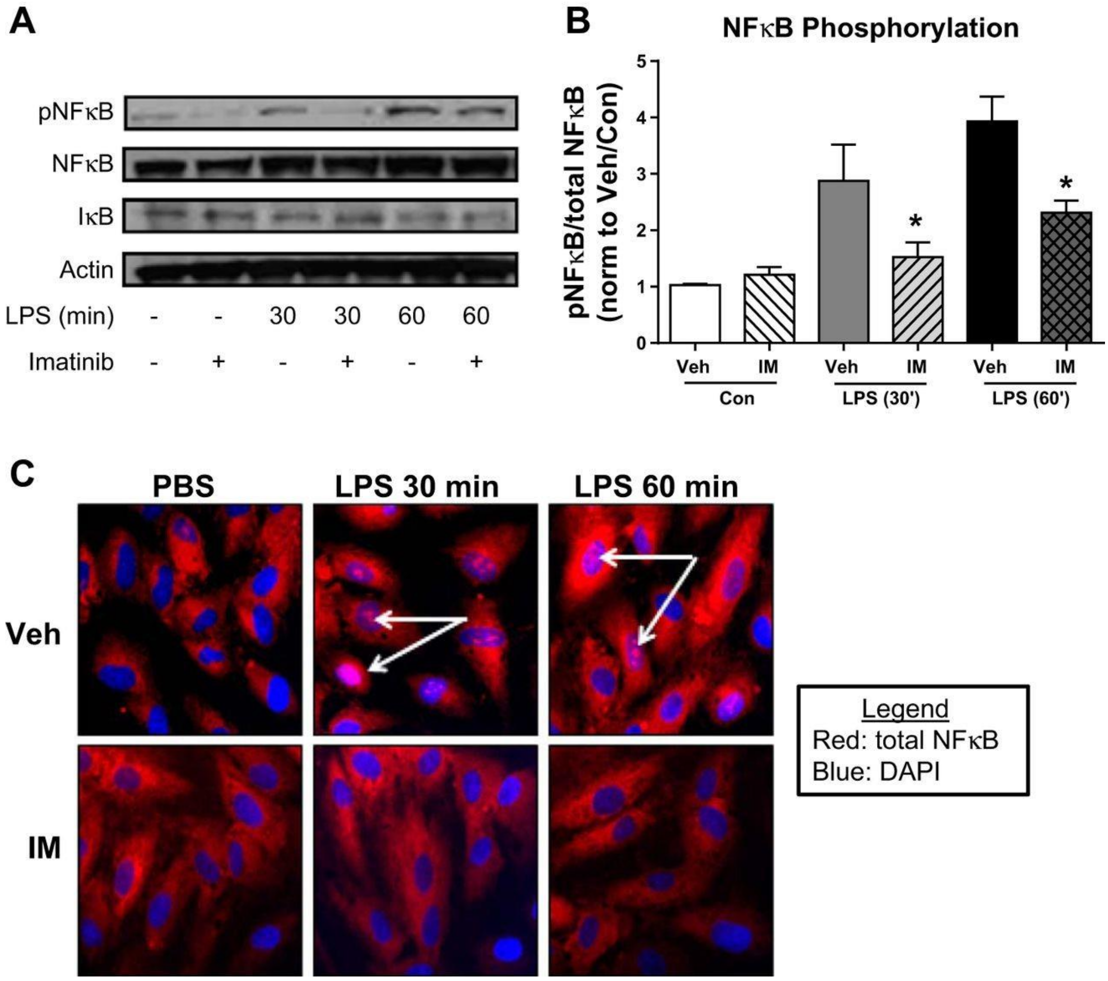


**Figure 6.** Imatinib inhibits LPS-induced NF-κB phosphorylation and nuclear translocation in vitro. Human pulmonary artery endothelial cells (HPAEC) were treated with imatinib (40 μM, 60 min) and then challenged with LPS (1 μg/ml, 0–60 min). Lysates were harvested for Western blots for phosphorylated NF-κB p65 (S536), total NF-κB p65 protein, IκB, and actin and quantified by densitometry (A and B). HPAEC plated on glass coverslips were subjected to identical conditions, and immunofluorescence microscopy was conducted to determine the localization of total NF-κB p65 protein (red). 4′,6-diamidino-2-phenylindole (DAPI) (blue) was used to stain the nuclei (white arrows) (C). Data are representative of 3 independent experiments. *$P < 0.05$ compared with nonchallenged samples.[20]

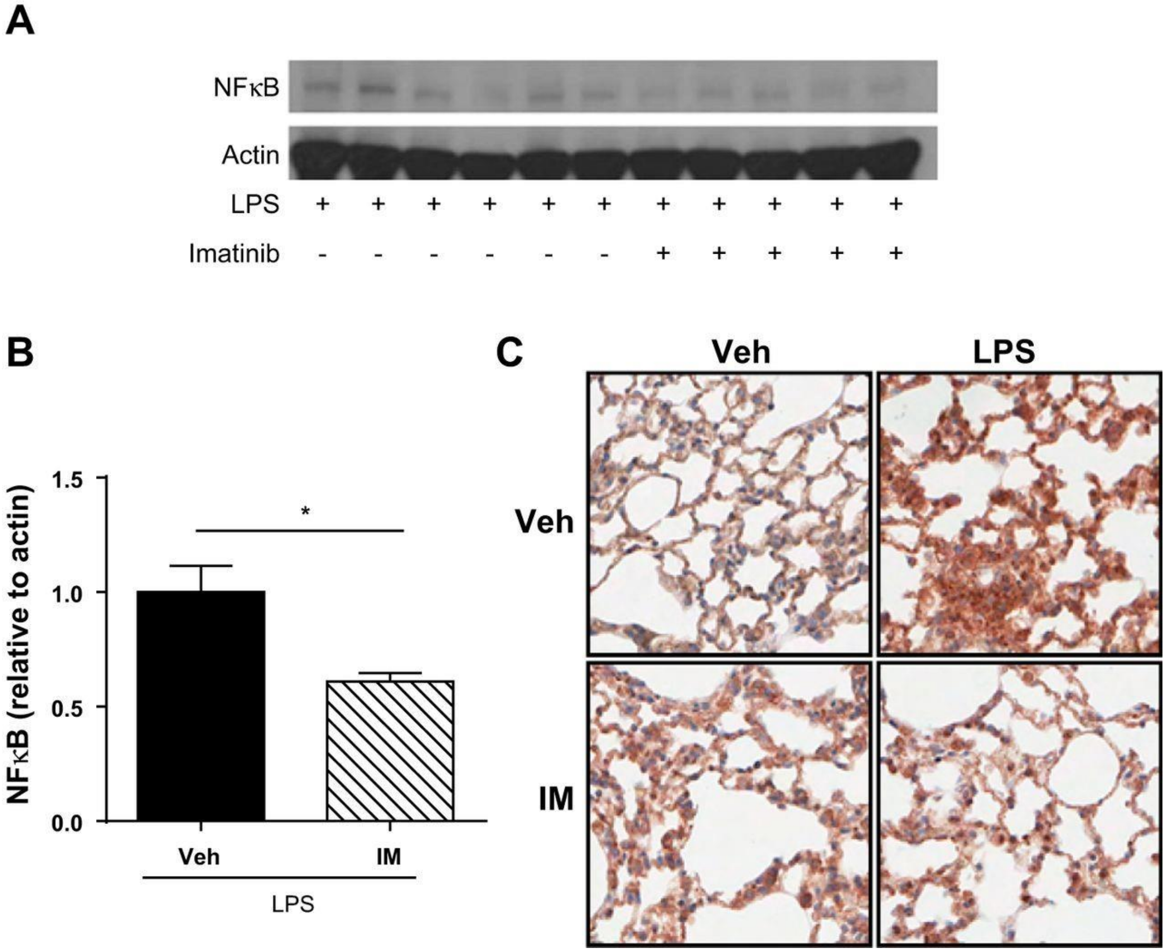


**Figure 7.** Imatinib decreases NF-κB expression in mouse lungs after LPS. Mice were challenged with LPS (1.0 mg/kg, it) (vs. PBS) and then received imatinib (75 mg/kg, ip) (vs. vehicle) 4 h later. Lung tissue homogenates were collected, subjected to Western blotting for total NF-κB p65 protein, and quantified by densitometry (A and B). Lanes in A represent lung homogenates from individual animals. Representative immunohistochemistry images are shown for NF-κB p65 taken by an individual blinded to experimental condition (C). *$P < 0.05$.[20]

It is important to address the clinical safety and availability of imatinib. Imatinib is clinically available worldwide and have been in clinical practice for several years with very well-known adverse event profiles. It is tolerable as single-agent for patients with hematological disorders.[22,23] Considering the half-life and other pharmacologic properties of imatinib, it is expected that the intended effect of the drug should be manifested by 14 days in this patient population.

## 1.6. Best Conventional Care

All subjects will receive the best conventional care (BCC) available according to local practice standards. Taking into account that practice standards will evolve as data from multiple ongoing studies become available, BCC may change overtime. In this study the BCC may include, but is not limited to: use of antipyretics, anti-infectives including antivirals, when approved, anti-inflammatory agents (e.g. chloroquine analogues), supplemental oxygen, non-invasive and invasive ventilation, vasopressor support, renal-replacement therapy, and ECMO.

Therapies that are shown to be effective but may not be licensed will be added as an exception to the exclusion criteria in order to allow for the most contemporary standard of care to include emergency use authorization treatments as they become available. Antivirals such as remdesivir will be permissible given the FDA authorized emergency use.

# 2. OBJECTIVES AND ENDPOINTS

## 2.1. Primary Objectives

To evaluate the efficacy and safety of oral administration of imatinib combined with BCC vs. placebo plus BCC in hospitalized patients with COVID-19

## 2.2. Primary Endpoint

The primary endpoint is the proportion of subjects with two-point improvement at Day 14 from baseline using the 8-category ordinal scale. The ordinal scale is an evaluation of the status at the first assessment of a given study day. This endpoint of clinical improvement has been used before in clinical trials in patients hospitalized with severe influenza[24] and with COVID-19[25] infection, and it is recommended by the WHO R&D Blueprint expert group[26].

The scale is as follows: 1) Not hospitalized, no limitations on activities; 2) Not hospitalized, limitation on activities and/or requiring home oxygen; 3) Hospitalized, not requiring supplemental oxygen – no longer requires ongoing medical care; 4) Hospitalized, not requiring supplemental oxygen - requiring ongoing medical care (COVID-19 related or otherwise); 5) Hospitalized, requiring supplemental oxygen; 6) Hospitalized, on non-invasive ventilation or high flow oxygen devices; 7) Hospitalized, on invasive mechanical ventilation or extracorporeal membrane oxygenation (ECMO); 8) Death.

## 2.3. Secondary Endpoints

- All-cause mortality at Day 28
- All-cause mortality at Day 60
- Time to a 2-point clinical improvement difference over baseline
- Duration of hospitalization
- Duration of ECMO or invasive mechanical ventilation (for subjects on ECMO of mechanical ventilation at Day 1)
- Duration of Intensive Care Unit (ICU) stay/requirement (for subjects in ICU at Day 1)
- Proportion of subjects with treatment-related serious adverse events
- Proportion of subjects who discontinue study treatment due to adverse events
- Time to SARS-CoV2 negative by reverse transcriptase-polymerase chain reaction (RT-PCR)
- Proportion of patients with a negative oropharyngeal or nasopharyngeal swab for SARS-CoV-2 by quantitative RT PCR on days 5, 10, 14, 21, and 28.

## 2.4. Exploratory Endpoints

- Determine the impact of treatment arms on IL-6 levels
- Obtain blood/PBMCs for storage to look at transcriptomics in severe disease
- Association of MHC with severity of illness
- Mean change in the ordinal scale from baseline
- Time to an improvement of one category from admission using an ordinal scale
- Duration of hospitalization

- Duration of new oxygen use
- Number of oxygenation free days
- Duration of new mechanical ventilation
- Number of ventilator free days

# 3. CRITERIA FOR STUDY SUBJECT ELIGIBILITY

## 3.1. Inclusion Criteria

Patients may be included in the study only if they meet **all** of the following criteria:

1. Ability to understand and willingness to sign a written informed consent document. Informed consent must be obtained prior to participation in the study. For patients who are too unwell to provide consent such as patients on invasive ventilator or ECMO, Legally Authorized Representative (LAR) can sign the informed consent.
2. Hospitalized patients $\geq$ 18 years of age
3. Positive RT-PCR assay for SARS-CoV-2 in the respiratory tract sample (oropharyngeal, nasopharyngeal or BAL) by Center for Disease Control or local laboratory within 7 days of randomization.
4. Women of childbearing potential must agree to use at least one primary form of contraception for the duration of the study.

## 3.2. Exclusion Criteria

Patients meeting any of the following criteria are **not** eligible for the study:

1. Patients receiving any other investigational agents in a clinical trial. Off-label use of agents such as hydroxychloroquine is not an exclusion criterion.
2. Pregnant or breastfeeding women.
3. Patients with significant liver or renal dysfunction function at screen as defined as:
   a. Direct bilirubin > 2.5 mg/dL
   b. AST, ALT, or alkaline phosphatase > 5 x upper limit of normal
   c. eGFR $\leq$ 30 mL/min or requiring renal replacement therapy
4. Patients with significant hematologic disorder at screen as defined as:
   a. Absolute neutrophil count (ANC) < 500/µL
   b. Platelet < 20,000/µL
   c. Hemoglobin < 7 g/dL
5. Uncontrolled undercurrent illness including, but not limited to, symptomatic congestive heart failure, unstable angina pectoris, uncontrolled active seizure disorder, or psychiatric illness/social situations that per site Principal Investigator's judgment would limit compliance with study requirements.
6. Known allergy to imatinib or its component products.
7. Any other clinical conditions that in the opinion of the investigator would make the subject unsuitable for the study.

## 3.3. Inclusion of Women and Minorities

Both men and women of all races and ethnic groups are eligible for this trial.

# 4. RECRUITMENT PLAN AND REGISTRATION PROCEDURES

Informed consent is a process that is initiated prior to the individual agreeing to participate in the study and continues throughout the individual's study participation. The informed consent form will be IRB-approved and the subject will be asked to read and review the document. Due to the excessive risk of COVID-19 transmission and to preserve limited personal protective equipment (PPE) for essential procedures, a **remote consenting process** will be used. The following considerations for remote informed consenting should be followed:

- The investigator and/or his designee is responsible for presenting the risks and benefits of study participation to the subject in simple terms using the informed consent document.
- Potential subjects will be given an informed consent form (either paper or electronic document in a portable electronic device) and allowed time to read this document.
- The elements of the informed consent, including discussion of the risk and benefits, will be done either over the phone or via remote video conferencing methods approved by the IRB.
- Research team member will document how the consent form was transmitted or given to participant, and what method was used to communicate the informed consent.
- The investigator will ensure that written informed consent is obtained from each subject or legally authorized representative by obtaining the appropriate signatures and dates on the informed consent document before the performance of protocol evaluations or procedures.
- Signatures may be obtained using the following options: (1) electronic signature, or (2) photograph of signature/signature page sent back to study team.
- Subjects must date the informed consent form to be valid.
- Study team member should document how signature was obtained.
-

If unable to obtain a participant signature on an informed consent document, the following should be followed:

- Document the method used for communication with the patient;
- Document means by which agreement was communicated;
- Document that no imaging technology was available to capture a signed consent form;
- A witness to the process AND a witness sign and date the informed consent form.

Participants must be informed that participation is voluntary and that they may withdraw from the study at any time, without prejudice. Copies of the informed consent documents will be given to the participants for their records. The informed consent process will be conducted and documented in the source document (including the date) before the participant undergoes any study-specific procedures. The rights and welfare of the participants will be protected by emphasizing to them that the quality of their medical care will not be adversely affected if they decline to participate in this study.

For patients who are too unwell to provide consent such as patients on invasive ventilator or ECMO, Legally Authorized Representative (LAR) can sign the informed consent.

At the time of consent, a unique patient ID number will be assigned to the subject by the Coordinating Center. If for some reason the subject rescreens, the same patient ID number will be assigned to the subject for rescreening.

Once the signed informed consent has been obtained and all pretreatment evaluations have been performed, eligibility of the patient will be reviewed by the treating physician. Only eligible patients will be enrolled on the study.

To register a patient, the following documents should be compiled by a member of their clinical team, scanned and emailed to the Lead Protocol Coordinator for eligibility verification:

- Signed informed consent form (ICF)
- Copy of required laboratory tests including chemistry, CBC, bilirubin, LFT, RT-PCR assay results, eGFR and evidence of negative pregnancy test results for eligible participants
- Completed Eligibility worksheet

If the patient is deemed eligible for enrollment, the Lead Protocol Coordinator will forward the completed packet to the Lead Data Manager (DM) and PI. The Lead DM and PI/co-Is will assign an enrollment number, the PI/co-Is determine stratification, register the patient on the study in OnCore, and approve the start of treatment. The approval will communicate back to the treating physician and IDS pharmacy by the Lead DM. No patients can start treatment on the study until their enrollment and treatment assignment is confirmed by email.

Following enrollment, patients should begin protocol treatment within 3 days. Issues that would cause treatment delays should be discussed with the Principal Investigator (Medical Monitor, Dr. Emadi). If a patient does not receive protocol therapy following registration, the patient's registration on the study may be canceled. The medical monitor and Lead DM should be notified of cancellations as soon as possible.

# 5. EXPERIMENTAL PLAN

## 5.1. Study Design

This is an individual patient-level, randomized, double-blind, placebo-controlled phase 3 clinical trial at the University of Maryland Medical System, MD. Eligible patients are randomly assigned in 1:1 ratio to receive either placebo or tyrosine kinase inhibitor imatinib for 14 days plus BCC. In both arms, all study participants receive the best available conventional supportive care according to local practice standards. Taken into account that there are many national and international studies are ongoing, the BCC may change overtime. In this study the BCC may include, but not limited to, antipyretics, anti-infectives including antivirals (e.g. remdesivir), anti-inflammatory agents (e.g. chloroquine analogues), supplemental oxygen, non-invasive and invasive ventilation, vasopressor support, renal-replacement therapy, and ECMO. In order to balance the severity of the respiratory illness between the two arms, per FDA recommendation randomization are stratified based on radiographic findings and oxygen requirements: 1) Severe disease: evidence of pneumonia on chest X-ray or CT scan OR chest auscultation (rales, crackles), and $SpO_2 \leq 92\%$ on ambient air or $PaO_2/FiO_2 < 300$ mmHg, and requires supplemental oxygen administration by nasal cannula, simple face mask, or other similar oxygen delivery device; 2) Critical disease: requires supplemental oxygen delivered by non-rebreather mask or high flow cannula OR use of invasive or non-invasive ventilation OR requiring treatment in an intensive care unit, use of vasopressors, extracorporeal life support, or renal replacement therapy.

In both arms, patients can receive concomitant antibiotics including azithromycin at the discretion of the treating physician as necessary. Patients with remain on the study for 60 days.

The trial is conducted in accordance with the principles of the Declaration of Helsinki and the Good Clinical Practice guidelines of the International Conference on Harmonization.

## 5.2. Study Drugs

Imatinib

All doses of imatinib should be administered with a meal and a large glass of water. Imatinib can be dissolved in water or apple juice for patients having difficulty swallowing. In this study, patients with confirmed COVID-19 positive tests receive imatinib for total of 14 days; 400 mg orally daily Days 1-14. Imatinib 400 mg tablets will be encapsulated using size 000 capsules and cellulose microcrystalline filler. For patients on ventilator or ECMO, imatinib will be given as oral suspension (40 mg/mL). To make the oral suspension, imatinib tablets will be crushed and mixed in Ora-sweet solution to yield a concentration of 40 mg/mL suspension by pharmacy. Additionally, in the absence of supportive microbiological testing results, we confirm that the in-use stability period for the prepared imatinib suspensions will be 24 hours at room temperature or 7 days at refrigerated conditions. Due to the COVID-19 crisis, healthcare professionals (nursing staff in particular) are faced with the major burden of caring for critically patients, such as those on ventilators or ECMO. We believe that it to be impractical to request nursing staff to prepare imatinib oral solution by immersing on the floor. Additionally, we also believe this practice could result in dosing errors if tablets are not fully dissolved and may compromise the medication during preparation. The local pharmacy staff will prepare imatinib oral suspension under hazardous hood with proper protected equipment, which is vented to outside and meet USP Pharmaceutical Compounding guidelines. Lastly, pharmacy staff will be educated regarding risk associated with crushing and handling of imatinib and required protective measures. The pharmacy staff will follow the American Society Health-System Pharmacists (ASHP) guidelines for handling hazardous drugs.

The PK of imatinib have been evaluated in studies in healthy subjects and in population PK studies in over 900 patients. Imatinib is well absorbed after oral administration with $C_{max}$ achieved within 2-4 hours post-dose. Mean absolute bioavailability of imatinib is 98%. There is no significant change in the PK of imatinib on repeated dosing, and accumulation is 1.5- to 2.5-fold at steady state when imatinib is dosed once-daily. At clinically relevant concentrations of imatinib, binding to plasma proteins in *in vitro* experiments is approximately 95%, mostly to albumin and α1-acid glycoprotein.[23]

For Warnings, Precautions, Drug interactions, and Geriatric use, see attached FDA Label for Imatinib dated 08/21/2018.

Placebo

The matching placebo will be packaged by Investigational Drug Service Pharmacy at University of Maryland Medical Center. The placebos will be prepared using size 000 capsules and cellulose microcrystalline filler. Imatinib 400 mg capsules and placebo capsules will be identical form and color. For patients on ventilator or ECMO, placebo will be given as oral suspension with similar process for making imatinib suspension.

Concomitant Medications/supportive care

In both arms, patients can receive concomitant available local standard of care antipyretics, antibacterials, antivirals, antifungals and anti-inflammatory including hydroxychloroquine at the discretion of the treating physician as necessary. For other drug-drug interaction particularly with CYP P450, the treating physician should consider risk and benefit of drug administration based on available information.

Co-administration of off-label immunomodulatory treatments for COVID-19 including but not limited to corticosteroids, sarilumab, clazakizumab, tocilizumab, and anakinra will be allowed

but may affect interpretability of study outcomes. The timing, dosing, and duration of these treatments will be meticulously collected, including any of these treatments that may be used for participants who experience progression of COVID-19 disease after study enrollment. Two analyses will be performed, the primary analysis will compare the primary endpoint in the two trial arms irrespective of any other treatment; the second analysis will be stratified for co-administration of immunomodulatory drugs.

### 5.3. Replacement of Subject

The primary analysis will be conducted using a strict intention to treat principle (ITT). All patients will be evaluable irrespective of the clinical course of their disease and the dose of study medication received.

### 5.4. Estimated Study Duration and End of Study

For an individual subject the length of participation is approximately 60 days. Per recommendation by the FDA, we extend the duration of follow-up to Day 60 to assess adverse events and mortality. FDA recommended that telephone visits (or telemedicine) could be considered if clinic visits are not feasible.

End of Trial is defined as the time when the last subject is assessed or receives an intervention for the purposes of final data collection for the study. End of study for individual subject is defined as the last day that protocol-specified assessments are conducted for an individual subject. End of treatment phase is defined as the last assessment for the protocol-specified treatment phase of the study for an individual subject. End of study (primary completion) is defined as when the last subject is assessed or receives an intervention for the purposes of final collection of data for the primary endpoint. End of follow-up is defined as when the last subject completes the last protocol-specified assessment in the study.

## 6. DOSE MODIFICATION AND STUDY DISCONTINUATION

### 6.1. Dose Modification and Interruption

Patients can remain on the study if they at least receive 80% of prespecified dosages of placebo or imatinib, i.e. patients may miss < 20% of the scheduled drug dosages for medical reason(s) (at the discretion of treating physician) or administrative reason(s) such as temporary unavailability of a drug for a specific dose/schedule or nursing/clerical errors. Patients remain in the ITT analysis irrespective of dosage received.

### 6.2. Study Discontinuation

Treatment with should be discontinued in the event of any of the following:

- Occurrence of Grade 4 AE at least probably related to imatinib/placebo.
- Occurrence of an AE which makes discontinuation from treatment necessary due to protocol-specified safety criteria or desirable in the investigator’s and/or the subject´s opinion
- Investigator’s decision that a change of therapy is in the subject´s best interest

- Intercurrent medical condition, which in the opinion of the investigator or the subject precludes further treatment of the subject
- Withdrawal of subject's consent to further study treatment
- Death
- Lost to follow-up

All reasons for treatment discontinuation will be documented in the CRFs. If a subject fails to keep the appointments for study visits, the investigator will document the reason and circumstances as completely and accurately as possible.

Subjects have the right to withdraw from the study at any time and for any reason without prejudice to his or her future medical care by the physician or at the institution.

Subjects can decline to continue receiving protocol-specified therapies or procedures at any time during the study but continue participation in the study. If this occurs, the Investigator is to discuss with the subject the appropriate processes for discontinuation from protocol-specified therapies and must discuss with the subject the options for continuation of the Schedule of Assessments and collection of data, including endpoints and AEs. The Investigator must document the change to the Schedule of Assessments and the level of follow-up that is agreed to by the subject (e.g., in person, through family/friends, in correspondence/communication with other physicians, from review of the medical records).

Withdrawal of consent for a study means that the subject does not wish to receive further protocol-specified therapies or procedures, and the subject does not wish to or is unable to continue further study participation. Subject data up to withdrawal of consent will be included in the analysis of the study, and where permitted, publically available data can be included after withdrawal of consent. The Investigator is to discuss with the subject appropriate procedures for withdrawal from the study. Per recommendation by FDA, multiple attempts will be made to obtain final outcomes on all subjects enrolled. The primary endpoint can be assessed without any diagnostic tests or imaging and we expect essentially all patients to be evaluable with respect to this endpoint. The only exception is in case a patient wants to withdraw from the study and demands that his/her data not be used. We expect this to be an exceptional situation.

# 7. STUDY ASSESSMENTS/PROCEDURES

## 7.1. Informed Consent and Demographics

All subjects must sign and date the most current IRB approved ICF. Confirmation that the ICF has been signed should occur before any study specific procedures are performed. All enrolled subjects should be re-consented with any updated versions of IRB approved informed consents during study participation as applicable and per institutional guidelines.

Demographic data that will be collected include sex, date of birth, race, and ethnicity to study their possible association with subject safety and treatment effectiveness.

The following assessments/procedures are to be completed during the screening period at time points designated in the Schedule of Assessments: Confirmation that the ICF has been signed, Eligibility confirmed based on inclusion/exclusion criteria, Medical history, Demographic data collection, Pregnancy test, Local laboratory assessments including CBC with differential and Chemistry and CRP and ECG, Subject Registration. Subsequent ECGs will be obtained if clinically indicated at the discretion of the treating physician. FDA recommends clinically indicated ECGs given the risk of QT prolongation and overlapping cardiac risk profiles with some or the concomitant medications.

## 7.2. Medical History

The Investigator or designee will collect relevant medical history before the start of study and with every AE reporting. All findings are recorded on the medical history CRF. Per FDA recommendation, the number of days between onset of symptoms and initiation of treatment will be collected.

## 7.3. General Physical Examination

Weight should be measured at every visit without shoes. If weight is not able to be obtained at the time of enrollment, self-reported weight is acceptable, including weight reported on patient's driver's license. The baseline physical examination will be a complete physical examination as much as clinical condition of the patients allows. The physical examination at subsequent study visits will consist of an interim examination to monitor for any changes from the baseline physical examination. Physical examination does not have to be comprehensive and is determined by the treating physician. For vital signs, the following measurements must be performed as outlined in the Schedule of Assessments: systolic/diastolic blood pressure, pulse rate, respirations and $O_2$ saturation, and temperature in intervals.

## 7.4. Laboratory Assessments

Analyses for all laboratory tests used throughout this study are listed in the Study Calendar. All screening and on-study laboratory samples will be collected and processed at the investigators local laboratory and analyzed locally. If for clinical reason(s) or isolation status laboratory sampling cannot be performed, it does not considered protocol deviation.

Urine or serum pregnancy tests will be performed locally on all females except for female subjects who are surgically sterile or > 2 years postmenopausal. If the pregnancy test is positive at screening the subject should not be enrolled. If a standard of care pregnancy test is collected during the study, and the result is positive, the treating physician should contact the principle investigator for instructions. If a female subject, or the partner of a male subject, becomes pregnant during the conduct of the study it must be reported on the UMB IRB Pregnancy Notification Worksheet.

## 7.5. Schedule of Assessments (Study Calendar)

| ASSESSMENTS/ OBSERVATIONS | Screen (D-3 to D1) | Day 1 | Day 2 | Day 5 | Day 8 | Day 10 | Day 14[a] | Day 21 | Day 28 | Day 60[b] |
|---|---|---|---|---|---|---|---|---|---|---|
| | | | | | | | ±2 d | ±2 d | ±3 d | ±7 d |
| | | | | | | | | | | |
| Informed consent | X | | | | | | | | | |
| Confirm eligibility | X | X | | | | | | | | |
| Randomization | | X | | | | | | | | |
| Study drug administration | | X | | | | | | | | |
| | | | | | | | | | | |
| Demographics | X | | | | | | | | | |
| Medical history | X | | | | | | | | | |
| Vital signs & Physical exam[d] | X | X | | X | | X | X | X | X | X |
| Clinical assessments[e] | X | X | | | | | | | | |
| CBC w diff count | X | | X | X | | X | X | | X | X |
| Chemistries[f] | X | | X | X | | X | X | | X | X |
| CRP | X | | | X | | | X | | X | X |
| ECG | X | | | | | | | | | |
| Pregnancy test | X | | | | | | | | | |
| AE assessment | | X | | | | | | | | |
| Concomitant medication | X | X | | X | | X | X | X | X | X |
| Primary endpoint assessment | | | | | | | X | | | |
| SARS-CoV-2 PCR[g] | | X | | X | | X | X | | X | |
| Correlative/scientific studies | | X | | X | | X | X | | X | X |

Screen (Day -3 to Day 1) - Screening and Day 1 visits can occur the same day.

[a] Early discharge visit - use Day 14 assessments
[b] Early termination visit - use Day 60 assessments
[c] For planned 14 days

[d] Clinically appropriate physical exams including vital signs and O2 saturation

[e] Clinical assessments - 7-point ordinal scale and respiratory treatment status. Daily while hospitalized.
[f] Chemistries include the following: sodium, potassium, chloride, bicarbonate, BUN, creatinine, glucose, calcium, magnesium, phosphorous, AST, ALT, total bilirubin, alkaline phosphatase, albumin, total protein, and LDH. If total bilirubin is elevated, then fractionated direct bilirubin can be measured. Amylase, lipase, coagulation factors, and uric acid are measured at the discretion of the investigators. Check D-dimer and ferritin levels when clinically indicated.

[g] Respiratory specimens (nasopharyngeal or oropharyngeal swabs)

# 8. REPORTING ADVERSE EVENTS / REGULATORY REQUIREMENTS

## 8.1. Definitions

**Adverse Event:** An AE is defined as any untoward medical occurrence in a clinical trial subject. The event does not necessarily have a causal relationship with study treatment. The Investigator is responsible for ensuring that any AEs observed by the Investigator or reported by the subject are recorded in the subject's medical record. If a new primary malignancy appears, it will be considered an AE.

**CTCAE term (AE description) and Grade:** The descriptions and grading scales found in the revised NCI Common Terminology Criteria for Adverse Events (CTCAE) version 5.0 will be utilized for AE reporting. All appropriate treatment areas should have access to a copy of the CTCAE version 5.0. A copy of the CTCAE version 5.0 can be downloaded from the CTEP web site.

**Attribution of the AE:**

- Definite – The AE is clearly related to the study treatment.
- Probable – The AE is likely related to the study treatment.
- Possible – The AE may be related to the study treatment.
- Unlikely – The AE is doubtfully related to the study treatment.
- Unrelated – The AE is clearly NOT related to the study treatment.

**The Definition of a Serious Adverse Event (SAE)**:
SAE is defined as any of the following:

- Any death that occurs while the patient is enrolled in the study including the follow-up period or within 30 days of completing the study
- Immediately life-threatening AE
- Requires inpatient hospitalization
- Prolongation of an existing hospitalization
- Congenital anomaly/birth defect
- Medically important event
- Disability/incapacity (persistent or significant)

## 8.2. Reporting of Adverse Events

The Principal Investigator is responsible for monitoring the safety of patients who have entered this study and for alerting the UMGCCC Data and Safety Monitoring and Quality Assurance Committee (DSM/QAC) as well as the FDA to any event that seems unusual, even if this event maybe considered an unanticipated benefit to the patient.

Any adverse experiences which occur at any time during the clinical study or within 30 days of receiving the last dose of study medication, whether or not related to the study drug, will be collected by the study team into the Case Report Forms and the site Institutional Review Board (IRB) per institutional policy.

The principal investigator is responsible for appropriate medical care of study participants during the study in connection with protocol procedures. After a study participant's completion of or discontinuation from the study, the investigator remains responsible to follow, through an

appropriate health care option, AEs that are serious or that caused the study participant to discontinue before completing the study.

## 8.3. Reporting of Serious Adverse Events

Any serious adverse experiences which occur at any time during the clinical study or within 30 days of receiving the last dose of study medication, whether or not related to the study drug, will be collected by the study team into the Case Report Forms and the site IRB as per institutional policy.

If the event meets the criteria for FDA mandatory IND safety reporting (serious + unexpected), the Coordinating Center will report the event to the FDA using the MedWatch form (FDA Form 3500A). Events which are assessed as "unexpected fatal or life-threatening" should be reported to FDA as soon as possible, but no later than 7 calendar days following the sponsor's initial receipt of the information. All other unexpected serious suspected adverse reactions suggesting significant risk to human subjects should be reported to FDA no later than 15 calendar days following the sponsor's initial receipt of the information.

The Principal Investigator is responsible for appropriate medical care of study participants during the study in connection with protocol procedures. After a study participant's completion of or discontinuation from the study, the investigator remains responsible to follow, through an appropriate health care option, AEs that are serious or that caused the study participant to discontinue before completing the study.

The procedure is as follows:

• All deaths should be reported immediately

• If the serious AE was unexpected and possibly, probably, or definitely related to the drug, the investigator or designee must initiate the reporting process within 24 hours of being made aware of the event.

• The FDA is to be informed of any serious AEs in accordance with established guidelines using the standard MedWatch forms (FDA Form 3500A).

## 8.4. Data Safety Monitoring Plan

It is the responsibility of the Principal Investigator to oversee the safety of the study. This safety monitoring will include careful assessment and appropriate reporting of AEs as noted above. This study will be monitored by the UMGCCC Data and Safety Monitoring and Quality Assurance Committee and will follow the Data Safety and Monitoring Plan as outlined in the Clinical Investigator Handbook of the UMGCCC Clinical Research Office. The DSMB will meet after each 20-30 patients are enrolled or every 3 months whichever comes sooner. Adverse event profile of imatinib is well known. Due to the dynamic environment of best conventional care, defined halting rules may not apply to future best conventional care processes Enrollment halting may occur by DSMB based on interim/cumulative data for evidence of study-related adverse events including anticipated toxicities, such as ECG parameters, Serious Adverse Events and laboratory parameters. At the official request of the DSMB the study can be unblinded. This decision will be discussed by the PI and other investigators as well as with the regulatory agencies including UMB IRB and FDA.

## 8.5. Compliance with Laws and Guidance

This study will be conducted in accordance with current U.S. Food and Drug Administration (FDA) Regulations, Good Clinical Practices (GCPs) and International Counsel on Harmonization (ICH) E6 Guidelines. The investigator and their sub-investigators will sign financial disclosure forms prior to participating in the study and one year post end of study (21 CRF 54.1-54.6).

## 8.6. Institutional Review Board (IRB)

This protocol is submitted to the University of Maryland IRB. Study procedures will begin once local IRB approval is secured. All amendments, instances of reportable new information (i.e. unanticipated problems, data breaches, etc.) and continuing review reports will be submitted to the University of Maryland IRB per institutional policy.

## 8.7. Clinical Trial Monitoring

Source Data verification monitoring will be conducted in accordance with the Greenebaum Comprehensive Cancer Center Sponsor-Investigator Monitoring Standard Operating Procedure (SOP), the Code of Federal Regulations (CFR), and FDA and International Counsel on Harmonization (ICH) E6 Guidelines. This will include central verification of eligibility of each patient enrolled and risk based monitoring strategies.

The Investigator and study staff are responsible for maintaining a comprehensive and centralized filing system of all study-related (essential) documentation, suitable for inspection at any time by representatives from UMGCCC and/or applicable regulatory authorities.

Elements to include are subject files containing informed consent forms and subject identification list, study files containing the protocol with all amendments, Investigator's Brochure, copies of pre-study documentation, and all correspondence to and from the IRB, Investigational product-related correspondence including Proof of Receipts (POR), Investigational Product Accountability Record(s), Return of Investigational Product for Destruction Form(s), Final Investigational Product Reconciliation Statement, as applicable. In addition, all original source documents supporting entries in the CRFs must be maintained and be readily available.

The Clinical Monitor is responsible for verifying the CRFs at regular intervals throughout the study to verify adherence to the protocol; completeness, accuracy, and consistency of the data; and adherence to local regulations on the conduct of clinical research. The Clinical Monitor is to have access to subject medical records and other study-related records needed to verify the entries on the CRFs.

The Investigator agrees to cooperate with the Clinical Monitor to ensure that any problems detected during these monitoring visits, including delays in completing CRFs, are resolved.

## 8.8. FDA Reporting

The FDA Annual Report will be submitted to the Agency within +/- 60 days of the date on the IND approval letter. Major amendments will be submitted to FDA at the time of IRB submission. Minor amendments will be reported to FDA at the time of the next major amendment or at the next FDA Annual Report (whichever comes first).

### 8.9. Records Retention

Essential documents should be retained until at least 2 years after the last approval of a marketing application in an ICH region and until there are no pending or contemplated marketing applications in an ICH region or at least 2 years have elapsed since the formal discontinuation of clinical development of the investigational product. Records and documents pertaining to the conduct of this study and the distribution of investigational drug, including CRFs, consent forms, laboratory test results, and medication inventory records will be maintained in secure offsite storage after completion of study follow-up and data analysis.

### 8.10. Compliance with Trial Registration and Results Posting Requirements

Under the terms of the Food and Drug Administration Modernization Act (FDAMA) and the Food and Drug Administration Amendments Act (FDAAA), the Sponsor of the trial is solely responsible for determining whether the trial and its results are subject to the requirements for submission to the Clinical Trials Data Bank, http://www.clinicaltrials.gov. Information posted will allow subjects to identify potentially appropriate trials for their disease conditions and pursue participation by calling a central contact number for further information on appropriate trial locations and trial site contact information.

### 8.11. Data Management

Source documents are original documents, data, and records from which the subject's CRF data are obtained. These include but are not limited to hospital records, clinical and office charts, laboratory and pharmacy records, diaries, microfiches, radiographs, and correspondence.

Clinical data will be entered into the OnCore® database either by remotely accessing OnCore® via Virtual Protected Network (VPN) or by the designated data manager at the University of Maryland Marlene and Stewart Greenebaum Comprehensive Cancer Center (UMGCCC). Oncore® is 21CRF11.10 (electronic medical records) compliant and is equipped for HIPAA compliant internet-based entry of protocol tracking and review information.

All study data will be collected by the research team at each and every study visit and recorded in the research record. This data will then be entered in to the OnCore® study database.

All source documents will be obtained and retained along with any study forms, and placed into the patient's research folder.

## 9. ADMINISTRATIVE AND LEGAL OBLIGATION

### 9.1. Protocol Amendments and Study Termination

If the PI or other investigators wish to amend the protocol, the IRB must be informed of all amendments and give approval. The Investigator must send a copy of the approval letter from the IRB to UMGCCC. Significant changes will be reviewed by the FDA as well.

The Investigator is to notify the IRB in writing of the study's completion or early termination.

## 9.2. Investigator Responsibilities for Data Collection

The Investigator is responsible to comply with the requirements for all assessments and data collection (including subjects not receiving protocol-specified therapies) as stipulated in the protocol for each subject in the study. For subjects who withdraw before completion of all protocol-required visits and are unable or unwilling to continue the Schedule of Assessments the Investigator can search publically available records [where permitted]) to ascertain survival status. This ensures that the data set(s) produced as an outcome of the study is/are as comprehensive as possible.

## 9.3. Language

CRFs must be completed in English. All written information and other material to be used by subjects and investigative staff must use vocabulary and language that are clearly understood.

## 9.4. Publication Policy

Authorship of any publications resulting from this study will be determined based on the following:

Authorship credit is to be based on (1) substantial contributions to conception and design, acquisition of data, or analysis and interpretation of data; (2) drafting the article or revising it critically for important intellectual content; (3) final approval of the version to be published; (4) Agreement to be accountable for all aspects of the work in ensuring that questions related to the accuracy or integrity of any part of the work are appropriately investigated and resolved.

When a large, multicenter group has conducted the work, the group is to identify the individuals who accept direct responsibility for the manuscript. These individuals are to fully meet the criteria for authorship defined above.

Acquisition of funding, collection of data, or general supervision of the research group, alone, does not justify authorship.

All persons designated as authors are to qualify for authorship, and all those who qualify are to be listed. Authors are required to meet standard ICMJE criteria.

Each author is to have participated sufficiently in the work to take public responsibility for appropriate portions of the content.

All publications (e.g., manuscripts, abstracts, oral/slide presentations, book chapters) based on this study must be submitted to Amgen for review. The Clinical Trial Agreement among the institution, Investigator, and Amgen will detail the procedures for, and timing of, Amgen's review of publication.

A description of the methodology and performance characteristics of the quantitative RT-PCR assay will be provided in the final study report.

## 9.5. Compensation

Any arrangements for compensation to subjects for injury or illness that arises in the study are described in the Informed Consent that is available as a separate document.

# 10. STATISTICAL CONSIDERATIONS

## 10.1. Randomization

The trial is designed as a placebo-controlled, double-blind, two-parallel arm, randomized trial with a uniform (1:1) allocation ratio to:

Arm A) Imatinib+BCC

Arm B) Placebo+BCC

Patients in both arms can receive the supportive care per local standards at the discretion of the treating physician. Randomization will be completed in the following manner: Centralized, concealed randomization will be executed by the UMGCCC Pharmacist. Data on eligible consented cases will be submitted electronically on the appropriate on-study form to the pharmacy, where the patient is randomized to Imatinib or placebo. The randomized treatment allocations use stratified, permuted block randomization with a variable block size; blocks are generated using a validated random number generator.

## 10.2. Primary endpoint

Proportion of participants who on Day 14, has experienced a 2-point or higher improvement in 8-category ordinal scale relative to their status score at the day of randomization (Day 0).

## 10.3. Sample size estimation and power

Group sample sizes of 102 in Arm A and 102 in Arm B achieve 80.6% power to detect a difference between the group proportions of 0.20. The proportion in Arm A (the imatinib treatment arm) is assumed to be 0.30 under the null hypothesis and 0.50 under the alternative hypothesis. The proportion in Arm B (the placebo control group) is 0.30. The test statistic used is the two-sided Fisher's Exact Test. The significance level of the test is targeted at 0.05. The significance level actually achieved by this design is $\alpha$=0.0385. The power of the test is calculated using binomial enumeration of all possible outcomes.

The sample size is not inflated for dropouts. All patients will be evaluable irrespective of the clinical course of their disease. The primary analysis will be conducted using an intention to treat principle (ITT) for participants who at least receive one dose of study drug or placebo. The sample size is not inflated for dropouts..

**<u>8-category ordinal scale</u>**

1) Not hospitalized, no limitations on activities
2) Not hospitalized, limitation on activities and/or requiring home oxygen
3) Hospitalized, not requiring supplemental oxygen – no longer requires ongoing medical care
4) Hospitalized, not requiring supplemental oxygen - requiring ongoing medical care (COVID-19 related or otherwise)
5) Hospitalized, requiring supplemental oxygen
6) Hospitalized, on non-invasive ventilation or high flow oxygen devices
7) Hospitalized, on invasive mechanical ventilation or extracorporeal membrane oxygenation (ECMO)
8) Death

## 10.4. Secondary endpoints

28-day overall survival; 60-day mortality, total length of stay in ICU; average time spent with invasive ventilation; duration of oxygen therapy; duration of hospitalization; and time from randomization to death; proportion of subjects with treatment-related SAE; proportion of subjects who discontinue study treatment due to adverse events.

## 10.5. Interim analysis

No interim analysis for efficacy is planned. All grade 4 and 5 toxicities will be reported within 24 hours to an independent DSMB. The DSMB will decide if early stopping due to unacceptable toxicity is necessary.

## 10.6. Safety profile

A full safety profile will be reported with severity graded according to CTCAE, attribution to treatment, duration and time to resolution of toxicity. The safety population consists of all individual who received at least one dose of study medication.

## 10.7. Analysis of Efficacy Endpoints

Efficacy endpoint will be analyzed using the 1-sided Fisher's Exact Test. Response rates and the difference in response rates between trial arms will be estimated with 95% confidence intervals.

# 11. CORRELATIVE SCIENTIFIC STUDIES

## 11.1. Viral Shedding

Viral shedding will be determined by testing eluate from oropharyngeal or nasopharyngeal swab using reverse transcription quantitative real time PCR (RT-q-PCR). The swabs will be stored at -80$^{o}$C until use. Each sample will be tested in duplicate according to established protocol for SARS-CoV-2 in a CLIA-certified laboratory. Per FDA recommendation, "we will consider conducting genotypic resistance testing to examine the ability of SARS-CoV-2 to develop resistance to HCQ when viral rebound is observed during the study. Regions including the receptor binding domain and the S1/S2 proteolytic cleavage sites in the S protein of SARS-CoV-2 may be of interest."

## 11.2. Peripheral blood collection

Approximately 30-50 ml of peripheral blood (if possible) at different time points per Study Calendar will be acquired by phlebotomy. PBMC will be isolated. The plasma will be stored at -80$^{o}$C. Cytokine response will be evaluated using Cytokine & Chemokine 34-Plex Human ProcartaPlex™ Panel 1A (ThermoFisher Scientific, Cat#EPX340-12167-901). A Luminex® 200 Instrument (LX-200) will be used to measure the cytokine concentration in plasma. Per FDA recommendation, we will try to determine SARS-CoV-2 RNA in collected blood samples.

## 11.3. Serology

Plasma will be used to determine the anti-SARS-Cov2 spike protein IgG and IgM levels by ELISA as well as IL-6. Purified spike protein will be coated in 96-well plates and incubated with serial dilution of patient plasma. As negative control plasma from pre-COVID-19 healthy individuals will be used. The antibodies will be detected using HRP-conjugated anti-hu-IgG and IgM antibodies followed by TMB reagent. The dilution at which the signal is reduced by 50% compared to maximum signal will be reported as $EC_{50}$.

# 12.REFERENCES


1. Chen N, Zhou M, Dong X, et al. Epidemiological and clinical characteristics of 99 cases of 2019 novel coronavirus pneumonia in Wuhan, China: a descriptive study. Lancet 2020;395:507-13.
2. Huang C, Wang Y, Li X, et al. Clinical features of patients infected with 2019 novel coronavirus in Wuhan, China. Lancet 2020;395:497-506.
3. World Health Organization (WHO). Coronavirus disease (COVID-19) Pandemic. https://wwwwhoint/emergencies/diseases/novel-coronavirus-2019 2020.
4. Liu K, Fang YY, Deng Y, et al. Clinical characteristics of novel coronavirus cases in tertiary hospitals in Hubei Province. Chin Med J (Engl) 2020.
5. Wang D, Hu B, Hu C, et al. Clinical Characteristics of 138 Hospitalized Patients With 2019 Novel Coronavirus-Infected Pneumonia in Wuhan, China. JAMA : the journal of the American Medical Association 2020.
6. Chapuy B, Panse M, Radunski U, et al. ABC transporter A3 facilitates lysosomal sequestration of imatinib and modulates susceptibility of chronic myeloid leukemia cell lines to this drug. Haematologica 2009;94:1528-36.
7. Gotink KJ, Broxterman HJ, Labots M, et al. Lysosomal sequestration of sunitinib: a novel mechanism of drug resistance. Clinical cancer research : an official journal of the American Association for Cancer Research 2011;17:7337-46.
8. Gotink KJ, Rovithi M, de Haas RR, et al. Cross-resistance to clinically used tyrosine kinase inhibitors sunitinib, sorafenib and pazopanib. Cell Oncol (Dordr) 2015;38:119-29.
9. Colombo F, Trombetta E, Cetrangolo P, et al. Giant Lysosomes as a Chemotherapy Resistance Mechanism in Hepatocellular Carcinoma Cells. PloS one 2014;9:e114787.
10. Ruzickova E, Skoupa N, Dolezel P, Smith DA, Mlejnek P. The Lysosomal Sequestration of Tyrosine Kinase Inhibitors and Drug Resistance. Biomolecules 2019;9.
11. Burger H, den Dekker AT, Segeletz S, et al. Lysosomal Sequestration Determines Intracellular Imatinib Levels. Molecular pharmacology 2015;88:477-87.
12. Fu D, Zhou J, Zhu WS, et al. Imaging the intracellular distribution of tyrosine kinase inhibitors in living cells with quantitative hyperspectral stimulated Raman scattering. Nat Chem 2014;6:614-22.
13. Dyall J, Coleman CM, Hart BJ, et al. Repurposing of clinically developed drugs for treatment of Middle East respiratory syndrome coronavirus infection. Antimicrobial agents and chemotherapy 2014;58:4885-93.
14. Garcia M, Cooper A, Shi W, et al. Productive replication of Ebola virus is regulated by the c-Abl1 tyrosine kinase. Sci Transl Med 2012;4:123ra24.
15. Coyne CB, Bergelson JM. Virus-induced Abl and Fyn kinase signals permit coxsackievirus entry through epithelial tight junctions. Cell 2006;124:119-31.
16. Reeves PM, Smith SK, Olson VA, et al. Variola and monkeypox viruses utilize conserved mechanisms of virion motility and release that depend on abl and SRC family tyrosine kinases. J Virol 2011;85:21-31.
17. Chilakapati SR, Serasanambati M, Vissavajjhala P, Kanala JR, Chilakapati DR. Amelioration of bleomycin-induced pulmonary fibrosis in a mouse model by a combination therapy of bosentan and imatinib. Exp Lung Res 2015;41:173-88.
18. Li M, Abdollahi A, Grone HJ, Lipson KE, Belka C, Huber PE. Late treatment with imatinib mesylate ameliorates radiation-induced lung fibrosis in a mouse model. Radiat Oncol 2009;4:66.
19. Wolf AM, Wolf D, Rumpold H, et al. The kinase inhibitor imatinib mesylate inhibits TNF-{alpha} production in vitro and prevents TNF-dependent acute hepatic inflammation. Proceedings of the National Academy of Sciences of the United States of America 2005;102:13622-7.
20. Rizzo AN, Sammani S, Esquinca AE, et al. Imatinib attenuates inflammation and vascular leak in a clinically relevant two-hit model of acute lung injury. Am J Physiol Lung Cell Mol Physiol 2015;309:L1294-304.
21. Gao J, Tian Z, Yang X. Breakthrough: Chloroquine phosphate has shown apparent efficacy in treatment of COVID-19 associated pneumonia in clinical studies. Biosci Trends 2020;14:72-3.

22. Schrezenmeier E, Dorner T. Mechanisms of action of hydroxychloroquine and chloroquine: implications for rheumatology. Nat Rev Rheumatol 2020;16:155-66.
23. FDA. Imatinib Label. Food and Drug Administration 2018:https://www.accessdata.fda.gov/drugsatfda_docs/label/2018/021588s053lbl.pdf.
24. Wang Y, Fan G, Salam A, et al. Comparative effectiveness of combined favipiravir and oseltamivir therapy versus oseltamivir monotherapy in critically ill patients with influenza virus infection. J Infect Dis 2019.
25. Cao B, Wang Y, Wen D, et al. A Trial of Lopinavir-Ritonavir in Adults Hospitalized with Severe Covid-19. N Engl J Med 2020.
26. WHO. Coronavirus disease (COVID-2019) R&D Blueprint. World Health Organization 2020:http://www.who.int/blueprint/priority-diseases/key-action/novel-coronavirus/en/.
